# MTERF3 Regulates Mitochondrial Ribosome Biogenesis in Invertebrates and Mammals

**DOI:** 10.1371/journal.pgen.1003178

**Published:** 2013-01-03

**Authors:** Anna Wredenberg, Marie Lagouge, Ana Bratic, Metodi D. Metodiev, Henrik Spåhr, Arnaud Mourier, Christoph Freyer, Benedetta Ruzzenente, Luke Tain, Sebastian Grönke, Francesca Baggio, Christian Kukat, Elisabeth Kremmer, Rolf Wibom, Paola Loguercio Polosa, Bianca Habermann, Linda Partridge, Chan Bae Park, Nils-Göran Larsson

**Affiliations:** 1 Max-Planck Institute for Biology of Ageing, Köln, Germany; 2 Department Laboratory Medicine, Karolinska Institutet, Stockholm, Sweden; 3 Helmholtz Center, Institute for Molecular Immunology, Munich, Germany; 4 Department of Biosciences, Biotechnologies, and Pharmacological Sciences, University of Bari Aldo Moro, Bari, Italy; 5 Institute for Medical Sciences, Ajou University School of Medicine, Suwon, Korea; 6 Mitochondria Hub Regulation Center, Dong-A University College of Medicine, Busan, Republic of Korea; University of Miami, United States of America

## Abstract

Regulation of mitochondrial DNA (mtDNA) expression is critical for the control of oxidative phosphorylation in response to physiological demand, and this regulation is often impaired in disease and aging. We have previously shown that mitochondrial transcription termination factor 3 (MTERF3) is a key regulator that represses mtDNA transcription in the mouse, but its molecular mode of action has remained elusive. Based on the hypothesis that key regulatory mechanisms for mtDNA expression are conserved in metazoans, we analyzed *Mterf3* knockout and knockdown flies. We demonstrate here that decreased expression of MTERF3 not only leads to activation of mtDNA transcription, but also impairs assembly of the large mitochondrial ribosomal subunit. This novel function of MTERF3 in mitochondrial ribosomal biogenesis is conserved in the mouse, thus we identify a novel and unexpected role for MTERF3 in coordinating the crosstalk between transcription and translation for the regulation of mammalian mtDNA gene expression.

## Author Summary

One of the main functions of the mitochondrial network is to provide the energy currency ATP to drive a large array of cellular metabolic processes. The formation of the mitochondrial respiratory chain, which allows this energy supply, is under the control of two separate genetic systems, the nuclear and the mitochondrial genomes, whose expressions have to be tightly coordinated to ensure efficient mitochondrial function. The regulation of mitochondrial genome expression is still poorly understood despite the profound importance of this process in human physiology, disease, and aging. Here, we make one step forward by unraveling a new role for the mitochondrial transcription termination factor 3 (MTERF3), which was initially characterized as a factor able to decrease mitochondrial transcription. Using gene invalidation approaches, we show in two distinct model organisms, the fruit fly and the mouse, that MTERF3 is not only involved in mitochondrial transcription but also favors the assembly of the mitochondrial ribosome and thereby reinforces the coordination between transcription and translation events, two key steps in mitochondrial genome expression.

## Introduction

There is a growing interest in molecular mechanisms regulating oxidative phosphorylation capacity because of the increasing number of diseases associated with mitochondrial dysfunction [1] and as aging is associated with mitochondrial functional decline [2], [3]. Regulation of mitochondrial gene expression has an important role in fine-tuning oxidative phosphorylation capacity because critical subunits of the respiratory chain and the ATP synthase are encoded by mitochondrial DNA (mtDNA) [4], [5]. The regulation of mtDNA expression is completely dependent on nuclear genes but the mechanisms are not fully understood [4]. The expression of mtDNA could, in principle, be controlled at many different levels, e.g. by regulation of mtDNA copy number, transcription initiation, mRNA stability, translation or stability of respiratory chain subunits. Mitochondrial transcription factor A (TFAM) is essential both for transcription initiation [6], [7], [8], [9] and mtDNA copy number control [10]. TFAM packages mtDNA into a compact protein-DNA structure termed the nucleoid [11], [12]. There is a good correlation between TFAM levels and mtDNA levels in eukaryotic cells and mtDNA cannot be stably maintained if not coated by TFAM. However, there are a large number of mtDNA molecules in any given cell and changes in copy number are a slow process that is unlikely to have a main regulatory importance. In support of this notion, experimental manipulation of TFAM expression has been used to create mouse models with moderate decrease or increase of mtDNA copy number, with no or only minor effects on oxidative phosphorylation capacity [10], [13].

The basal mitochondrial transcription machinery consists of the nuclear-encoded mitochondrial RNA polymerase (POLRMT), TFAM and mitochondrial transcription factor B2 (TFB2M), which together are sufficient and necessary for transcription initiation *in vitro* [6], [14], [15], [16]. A large number of nucleus-encoded proteins have been reported to directly interact with and modulate the activity of the basal mitochondrial transcription machinery [17], [18], but this whole area is lacking a consensus for the role of these putative intramitochondrial transcription factors [14].

We have recently demonstrated that the mammalian mitochondrial leucine-rich pentatricopeptide repeat containing (LRPPRC) protein [19] and its fly homolog the bicoid stability factor (BSF) protein [20] are essential and have very similar roles in controlling mRNA stability, mRNA polyadenylation and coordination of translation in metazoan mitochondria [19], [20]. Regulation of mitochondrial translation not only involves mRNA maturation and stability, but also factors regulating translation and ribosomal biogenesis [21], [22]. An example of these factors is the adenine dimethyltransferase TFB1M, which modifies the 12S rRNA of the small ribosomal subunit and is necessary for the stability of the small ribosomal subunit and ribosomal biogenesis [23].

The role of the MTERF (mitochondrial transcription termination factor)-family of proteins [24] in regulation of mtDNA expression is of particular interest, because its members have been reported to influence mtDNA expression at different levels. MTERF1 has been suggested to play a role in mitochondrial transcription termination, by binding mtDNA downstream of the two mitochondrial rRNA genes to regulate the ratio between transcription of the upstream rRNA genes and the downstream mRNA genes [25], [26], [27], [28], [29]. In addition, MTERF1 has been reported to have a role in activating mtDNA transcription [30]. Mice lacking the *Mterf2* gene are viable, but have been reported to develop myopathy and memory deficits [31]. The exact molecular mechanisms of MTERF2 function remain unclear, but it has been reported to bind the mitochondrial promoter region and to stimulate transcription initiation [31], whereas another report showed that MTERF2 associates with nucleoids without displaying sequence-specific DNA binding [32]. MTERF3 and MTERF4 are both essential for embryonic survival [5], [33]. Characterization of conditional knockout mice has shown that MTERF3 functions as a negative regulator of mtDNA transcription initiation by interacting with the control region to inhibit activation of the two mitochondrial promoters [5]. Loss of MTERF3 in the mouse heart leads to a massive activation of mtDNA transcription and a severe respiratory chain deficiency, possibly caused by imbalanced amounts of mtDNA transcripts [5]. MTERF4 forms a heterodimer with the cytosine methyltransferase NSUN4 and targets this enzyme to the large ribosomal subunit [33], [34], where it likely modifies 16S rRNA to regulate mitochondrial ribosomal biogenesis.

The mitochondrial genomes of flies and mammals have the same gene content although there are differences in gene order and expression patterns [35], [36]. This high level of conservation of metazoan mtDNA suggests that important regulators of mtDNA expression also may be conserved. We therefore decided to use a cross-species comparison approach to further study the *in vivo* role of MTERF3. We demonstrate here that knockout and knockdown of the *Mterf3* gene expression in *Drosophila melanogaster* leads to activation of mtDNA transcription and impaired mitochondrial translation. We further show that imbalanced transcription is not the only cause of the altered mtDNA expression because also the 16S rRNA levels are reduced and the assembly of the large (39S) mitochondrial ribosomal subunit is impaired. These findings prompted us to reinvestigate the role for MTERF3 in the mouse, where we also found a reduction in levels of the 39S mitochondrial ribosomal subunit and impaired ribosomal assembly in the absence of MTERF3. These findings identify a novel role for MTERF3 in the biogenesis of metazoan mitochondrial ribosomes and point to a close crosstalk between transcription initiation and ribosomal biogenesis in control of mtDNA expression and regulation of oxidative phosphorylation capacity.

## Results

### DmMTERF3 is a conserved mitochondrial protein

We performed an extensive phylogenetic analysis of *Mterf3* and found a single gene ortholog in *Drosophila melanogaster*, which we denoted *DmMterf3* (Figure S1A). We used algorithms to predict the subcellular localization for the DmMTERF3 protein and found a high probability for mitochondrial localization using either Mitoprot (0.986) or TargetP (0.875) softwares. Next, we performed live imaging of cells expressing GFP-tagged DmMTERF3 after counterstaining with MitoTracker Deep Red (Figure S1B) and found a co-localization rate of 94.9±1.4% in Schneider (S2R+) cells (n = 8 analyzed cells) and 98.3±0.4% in HeLa cells (n = 10), thus experimentally verifying the predicted mitochondrial localization of DmMTERF3.

### Knockout of *DmMterf3* in flies leads to mitochondrial dysfunction and increased steady-state levels of mitochondrial transcripts

**Figure 1:**
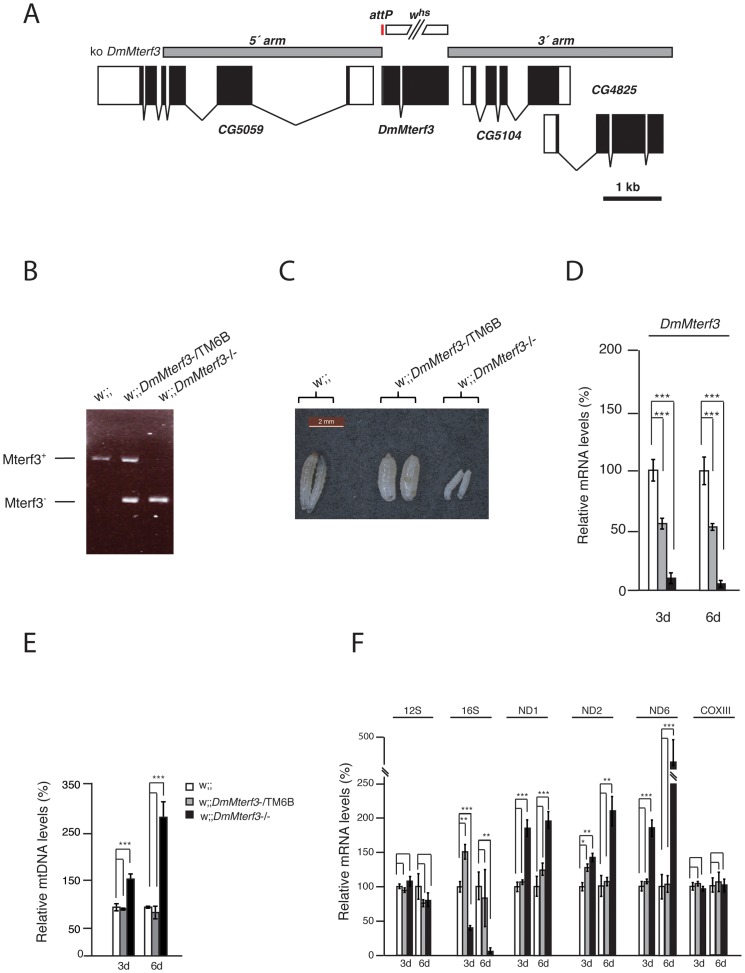
Creation and characterization of *DmMterf3* knockout larvae. (A) The *DmMterf3* locus and generation of a *DmMterf3* null mutant. *DmMterf3* is located on the third chromosome at cytological position 77C6. The construct (ko *DmMterf3*) used for ends-out homologous recombination is indicated by grey boxes, coding sequences in exons are indicated by black boxes and non-coding sequences in exons by white boxes. The gap between grey boxes represents the genomic region replaced by attP and a *white* marker gene. The white marker gene was subsequently removed by crossing to *cre*-recombinase expressing flies. (B) PCR analysis of wild-type and knockout alleles for *DmMterf3*. (C) Body size comparison of *DmMterf3* knockout larvae 6 days after egg-laying (ael) showing reduced size. (D) QRT-PCR analysis of *DmMterf3* transcript levels in control (white and grey bars) and *DmMterf3* KO larvae (black bars) at 3 and 6 days ael. (E) Q-PCR analysis of mtDNA levels in larvae at 3 and 6 days ael. (F) QRT-PCR analysis of steady-state levels of mitochondrial mRNAs normalized to the nuclear ribosomal protein 49 transcript levels in larvae at 3 and 6 days ael. Error bars indicate mean ± SEM (^*^p<0.05; ^**^p<0.01; ^***^p<0.001; n = 5).

In order to analyze the *in vivo* function of *DmMterf3* we generated knockout flies by ends-out homologous recombination [37] to replace the complete coding sequence for *DmMterf3* with an attP-site and a loxP-flanked marker gene denoted *white* (Figure 1A). Heterozygous knockout flies (genotype *DmMterf3* ^+;white^) were crossed to *cre*-recombinase expressing flies to remove the *white* gene and thereafter the third chromosome balancer *Tubby* (TM6B) was introduced. This balancer chromosome causes the Tubby larval phenotype, which will segregate with the wild-type *DmMterf3* allele in our crosses. The homozygous removal of *DmMterf3* as well as the excision of *white* was confirmed by PCR analysis of genomic DNA with gene-specific primers (Figure 1B). *DmMterf3* knockout (*DmMterf3* ^−/−^) larvae have a profoundly reduced body size and die in the third instar larval stage, whereas heterozygous *DmMterf3* ^+/−^ larvae pupate and develop into flies in a similar manner as wild-type larvae (Figure 1C). Quantitative reverse transcription (qRT)-PCR from *DmMterf3* ^−/−^ larvae showed ∼90% reduction of the *DmMterf3* transcript levels at 3 days after egg-laying (ael) and ∼95% reduction at 6 days ael (Figure 1D). The residual levels of *DmMterf3* transcript found in *DmMterf3* ^−/−^ larvae at 3 days ael are most likely due to a persisting maternal contribution because we saw further reduction of *DmMterf3* transcript levels in older *DmMterf3* ^−/−^ larvae (Figure 1D). Loss of DmMTERF3 resulted in increased mtDNA levels in knockout larvae at 3 and 6 days ael (Figure 1E) and an increase of ND1, ND2 and ND6 steady-state transcript levels, whereas the steady-state levels of the COXIII and 12S rRNA transcripts were unchanged and levels of the 16S rRNA profoundly reduced (Figure 1F).

To summarize, there are important phenotypic similarities between *DmMterf3* knockout flies and *Mterf3* knockout mice [5], because in both cases the gene is essential and its inactivation leads to increased steady-state levels of most mitochondrial transcripts, as well as reduction of 16S rRNA transcript levels. The early death of *DmMterf3* ^−/−^ larvae prevented a detailed molecular characterization of the phenotype and we therefore proceeded to use *DmMterf3* RNAi flies for the subsequent studies.

### Knockdown of *DmMterf3* expression causes reduced larval size and lethality at the pupal stage

We proceeded to use a UAS-GAL4 based strategy to knock down *DmMterf3* expression in flies. We first tested the RNAi construct by using it in conjunction with the eye-specific *eyeless*-GAL4 driver and found a massive phenotype with reduced eye-size and disorganized head structure consistent with efficient silencing of *DmMterf3* expression (Figure S2A, S2B).

Next, we proceeded with the ubiquitous knockdown (KD) of *DmMterf3* expression using the *daughterless*-GAL4 driver (da-GAL4) to produce a KD line containing transgenes encoding both the da-GAL4 transactivator and the inducible UAS-RNAi construct w;;UAS-*DmMterf3*-RNAi/da-GAL4. We also generated two control lines, the first line w;;da-GAL4/+ only containing the da-GAL4 transgene and the second line w;;UAS-*DmMterf3*-RNAi/+ only containing the transgene encoding the inducible RNAi construct. The KD line and the two control lines were analyzed in parallel for all subsequent experiments.

**Figure 2:**
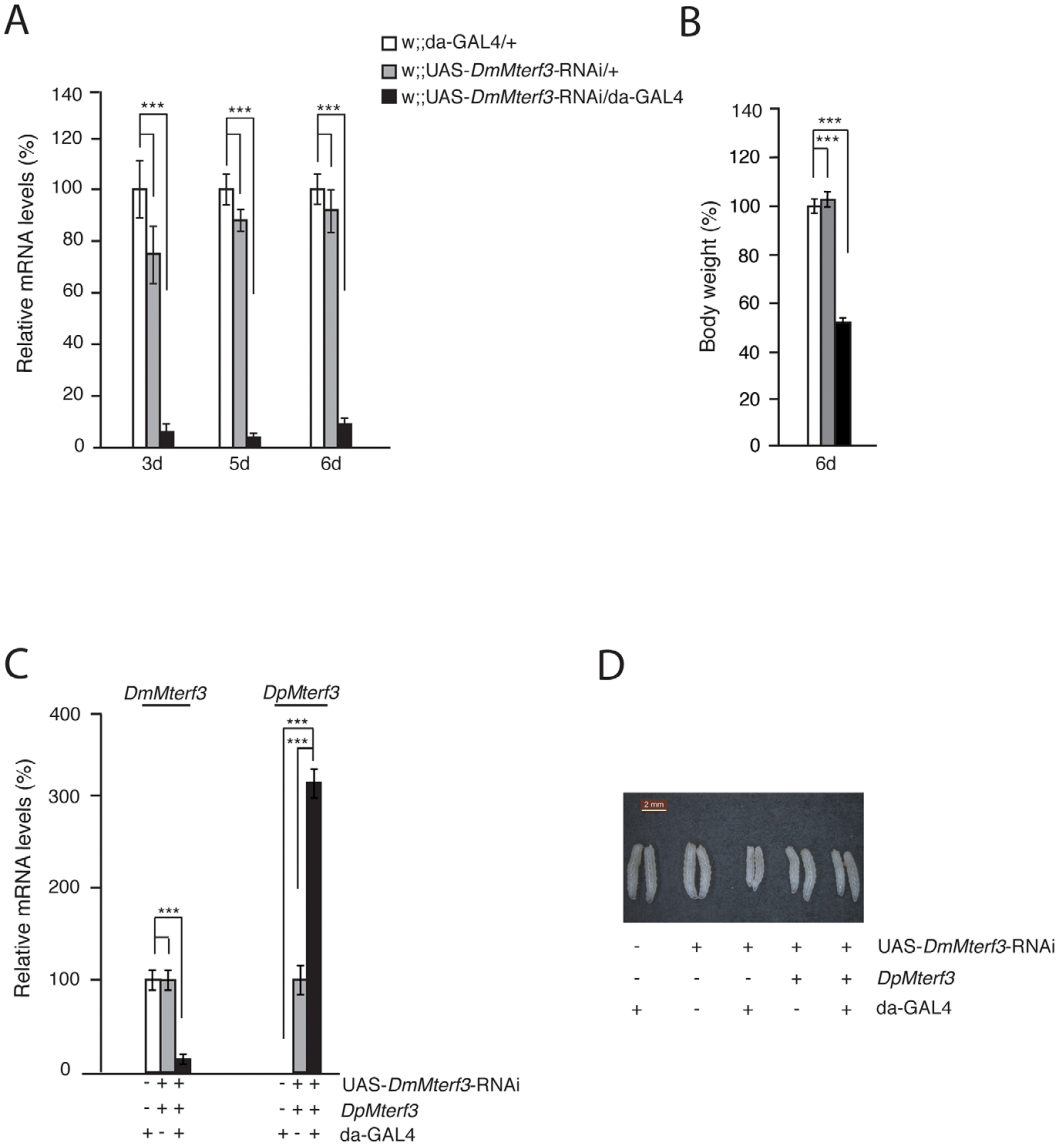
Phenotype and molecular characterization of *DmMterf3* knockdown larvae. (A) QRT-PCR analysis of DmMterf3 transcript levels in control (white and grey bars) and DmMterf3 KD larvae at 3, 5 and 6 days ael (black bars). (B) Body weight of control and *DmMterf3* KD larvae at 6 days ael. (C) QRT-PCR analysis of *DmMterf3* and *DpMterf3* transcript levels in *DmMterf3* KD and rescue larvae at 6 days ael (black bars). (D) Body size comparison of larvae that are *DmMterf3* KD or *DmMterf3* KD with *DpMterf3* rescue. The rescued larvae are indistinguishable from controls. Error bars indicate mean ± SEM (*p<0.05; **p<0.01; ***p<0.001; n = 5).

Ubiquitous knockdown of *DmMterf3* expression led to ∼80–90% reduction of *DmMterf3* transcript levels in KD larvae at 3, 5 and 6 days ael (Figure 2A). The *DmMterf3* KD larvae were visibly smaller from 4 days ael and onwards, as documented by a reduced body weight in comparison with controls (Figure 2B). Eventually, *DmMterf3* KD larvae displayed delayed larval development and died at the pupal stage.

In order to rule out off-target RNAi effects, we generated a transgenic fly line expressing *Drosophila pseudoobscura* (Dp) MTERF3. DpMTERF3 has ∼80% similarity to DmMTERF3 at the amino acid level, whereas the nucleotide sequence of the corresponding genes differs substantially (Figure S3A, S3B). We therefore hypothesized that the RNAi construct directed against *DmMterf3* expression would have no effect on *DpMterf3* expression and that the DpMTERF3 protein therefore would be expressed to rescue the lethal *DmMterf3* KD phenotype. This prediction was indeed confirmed and qRT-PCR analysis showed loss of *DmMterf3* transcripts and the presence of the *DpMterf3* transcript in rescued flies (Figure 2C). Importantly, expression of DpMTERF3 fully rescued the growth phenotype of *DmMterf3* KD larvae (Figure 2D), which indicates the absence of off-target effects of the RNAi construct we are using.

### Loss of DmMTERF3 causes a progressive respiratory chain deficiency

**Figure 3:**
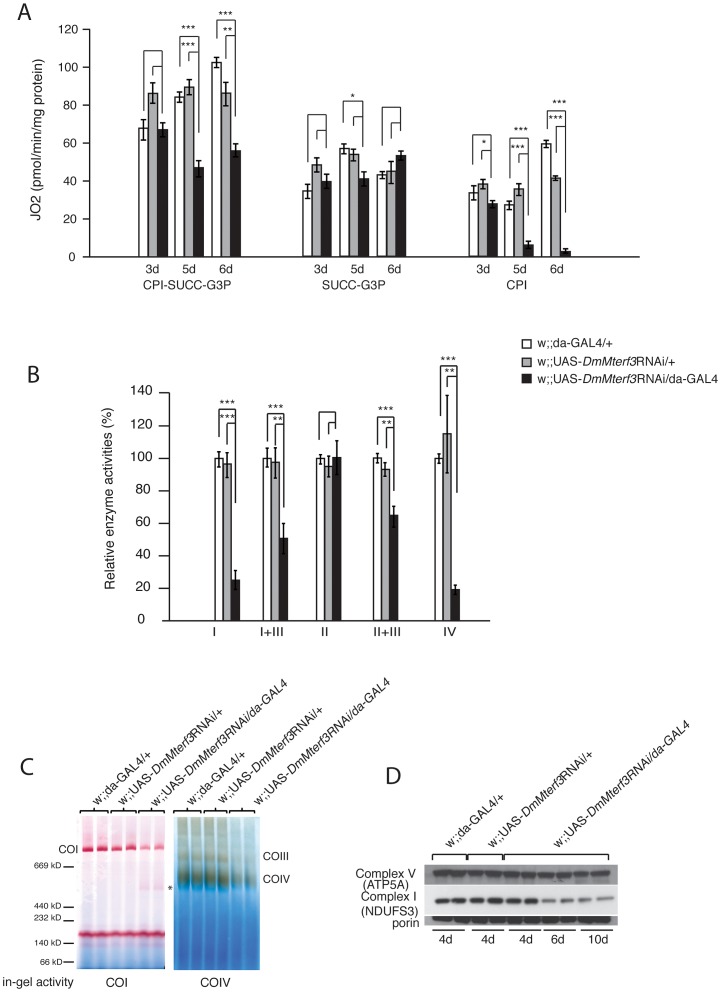
Biochemical measurement of respiratory chain function. (A) Oxygen consumption normalized to protein content in permeabilized control (white and grey bars) and *DmMterf3* KD (black bars) larvae at 3, 5 and 6 days ael. Substrates that are metabolized to deliver electrons entering at the level of complex I (CPI), complex II (SUCC) and/or glycerol-3-phosphate dehydrogenase, (G3P) were used (n = 3–7). (B) Relative enzyme activities of respiratory chain enzyme complex I (NADH coenzyme Q reductase), complex I+III (NADH cytochrome c reductase), complex II (succinate dehydrogenase), complex II+III (succinate cytochrome c reductase) and complex IV (cytochrome c oxidase) in control (white and grey bars) and *DmMterf3* KD larvae at 6 days ael (black bars) (n = 6–7). (C) BN-PAGE analysis and in-gel activity of complex I and complex IV in mitochondrial protein extracts from control and *DmMterf3* KD larvae 6 days ael. (D) Western blot analysis using antibodies against the nuclear-encoded NDUFS3 subunit of complex I and the α-subunit of ATP synthase (complex V) in control larvae at 5 days ael and in *DmMterf3* KD larvae at 5, 6 and 10 days ael. Error bars indicate mean ± SEM (^*^p<0.05; ^**^p<0.01; ^***^p<0.001).

We proceeded to investigate the biochemical consequences of reduced *DmMterf3* expression by measuring mitochondrial respiratory chain capacity in permeabilized tissue extracts from larvae. *DmMterf3* KD larvae at 3–6 days ael showed a major reduction in the oxygen consumption rates in the presence of substrates that are metabolized to deliver electrons to the respiratory chain at the level of complex I (CPI) or complex I and II (CPI-SUCC-G3P) (Figure 3A). In contrast, substrates metabolized to deliver electrons at the level of complex II or glycerol-3-phosphate dehydrogenase, thereby eliciting electron transport by-passing complex I, had no major effect on oxygen consumption (Figure 3A). We also measured the activities of individual respiratory chain complexes in larvae at 6 days ael and found severely decreased enzyme activities of all complexes containing mtDNA-encoded subunits in KD larvae, whereas the exclusively nucleus-encoded complex II was unaffected (Figure 3B).

Additionally, we assessed the levels of assembled respiratory chain enzyme complexes by Blue-Native polyacrylamide gel electrophoresis (BN-PAGE). Assembled complex I and IV were markedly reduced in *DmMterf3* KD larvae at 6 days ael, as indicated by a reduction of complex I and IV in-gel activity (Figure 3C and Figure S4B). In addition, a smaller and partially assembled form of complex I was present in *DmMterf3* KD larvae at 6 days ael, again indicating a problem with complex I (Figure 3C, asterisk). Western blot analysis showed low levels of the NDUFS3 subunit of complex I, whereas the levels of the ATP5A subunit of complex V (ATP synthase) were unaffected in KD larvae at 6 days ael (Figure 3D) and in knockout larvae at 3 days ael (Figure S4A). Taken together, these results show that complex I and IV are the most affected of the oxidative phosphorylation complexes in the absence of DmMTERF3.

### Increased steady state-levels of mtDNA and most mitochondrial transcripts in *DmMterf3* KD larvae

**Figure 4:**
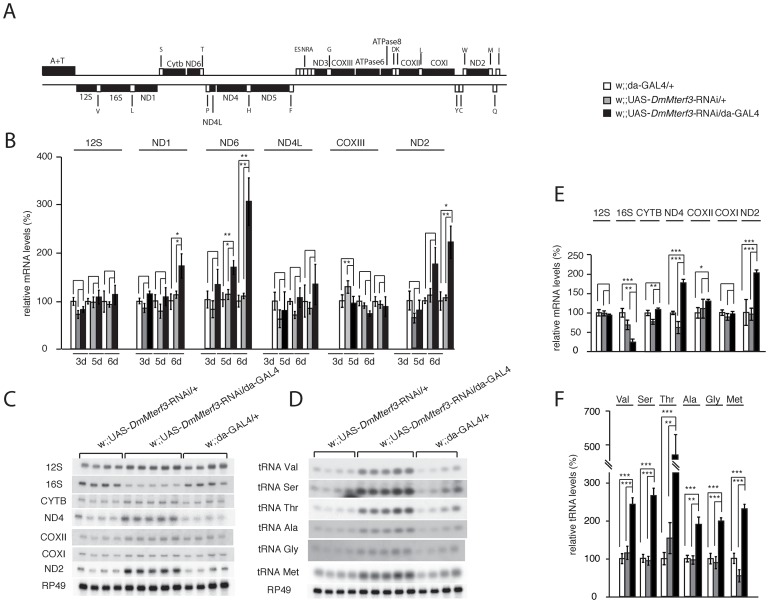
Steady-state levels of mtDNA and mitochondrial transcripts. (A) Schematic linearized map of the mitochondrial genome of *Drosophila melanogaster*. (B) QRT-PCR analysis of steady-state levels of mitochondrial mRNAs in control and *DmMterf3* KD larvae at 3, 5 and 6 days ael. The analysis was normalized to levels of the transcript encoding the nuclear ribosomal protein 49. (C) Northern blot analysis of steady-state levels of mitochondrial rRNAs and mRNAs from control and *DmMterf3* KD larvae 6 days ael. Loading of gels was normalized to the transcript encoding the nuclear ribosomal protein 49. (D) Northern blot analysis of steady-state levels of mitochondrial tRNAs in control and *DmMterf3* KD larvae 6 days ael. (E) Quantification of steady-state levels of mitochondrial rRNAs and mRNAs in control and *DmMterf3* KD larvae at 6 days ael. Loading of gels was normalized to the transcript encoding the nuclear ribosomal protein 49. (F) Quantification of steady-state levels of mitochondrial tRNAs in control and *DmMterf3* KD larvae at 6 days ael. Error bars indicate mean ± SEM (^*^p<0.05; ^**^p<0.01; ^***^p<0.001. n = 4–5).

The progressive respiratory chain dysfunction induced by loss of DmMTERF3 (Figure 3) led us to investigate mtDNA levels and mtDNA expression (Figure 4 and Figure S5A). Similar to what we observed in *DmMterf3* knockout embryos (Figure 1E), we found an increase of mtDNA levels in *DmMterf3* KD larvae at 6 days ael (Figure S5A), possibly caused by a compensatory activation of mitochondrial biogenesis as previously observed in respiratory chain deficient flies [20] and mice [23].

We proceeded to use qRT-PCR to analyze levels of mtDNA-encoded transcripts (Figure 4A, 4B) in KD larvae at 3, 5 and 6 days ael. We observed a progressive increase in levels of the ND1, ND2 and ND6 transcripts, whereas there were no changes in the levels of ND4L, COXIII and 12S rRNA transcripts (Figure 4B). We also used Northern blots to analyze transcript steady-state levels in larvae at 6 days ael and found an increase of the ND2, ND4 and Cytb transcripts, whereas the levels of COXI, COXII and 12S rRNA transcripts were unaltered (Figure 4C, 4E). We observed decreased levels of the 16S rRNA (Figure 4C, 4E and Figure S5B, S5C). In contrast, all tRNAs analyzed, regardless of the location of the corresponding genes in the genome, showed a progressive increase of their steady-state levels (Figure 4D, 4F and Figure S5D, S5E). Interestingly, the mtDNA transcript profiles in *Mterf3* knockout mice [5] and *DmMterf3* KD fly larvae show many similarities, including increased levels of many, but not all, mRNAs, increased levels of tRNAs and decreased levels of the 16S rRNA.

**Figure 5:**
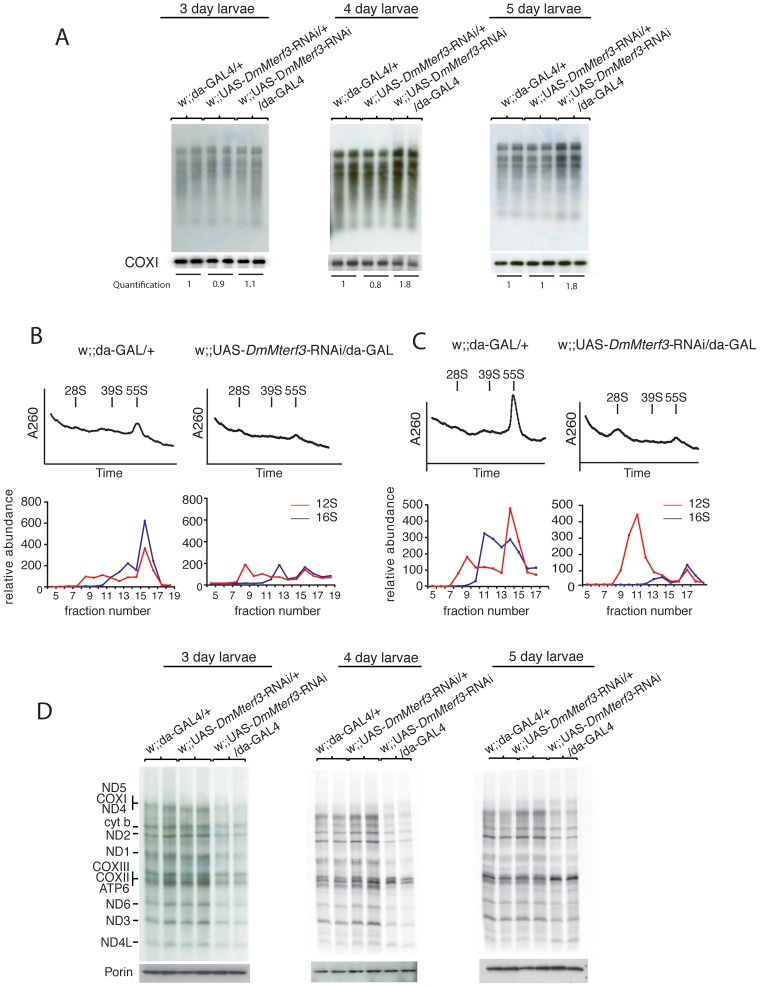
Mitochondrial *de novo* transcription, ribosome assembly, and *de novo* translation. (A) *In organello* transcription assays in isolated crude mitochondrial preparations from larvae at 3, 4 and 5 days ael. The upper panels show the *de novo* transcription of mtDNA and the lower panels show Northern blot analysis to detect COX I transcript as loading controls. *De novo* transcription was quantified for each lane and normalized to COX I mRNA steady-state levels, each value correspond to the average of each replicate. (B) Sedimentation analysis of ribosome assembly in control and *DmMterf3* KD larvae at 3 days ael. The small (28S) ribosomal subunit, the large (39S) ribosomal subunit and the assembled (55S) ribosome are indicated by arrows. Fractions were collected with continuous monitoring of absorbance at 260 nm (upper panels) and subsequently analyzed by qRT-PCR to detect 12S (red) and 16S (blue) rRNA transcripts (lower panels). (C) Sedimentation analysis of ribosome assembly in control and *DmMterf3* KD larvae at 5 days ael analyzed as in (B). (D) Analysis of mitochondrial *de novo* translation as determined by S^35^-methionine incorporation into newly synthesized proteins *in organello* in control and *DmMterf3* KD larvae at 3, 4 and 5 days ael. Equal loading was assessed by analyzing an aliquot of the mitochondrial protein extract to be used for incubation with S^35^-methionine on a SDS-PAGE gel followed Western blotting to detect Porin.

We have previously observed that the steady-state levels of tRNAs, but not those of mRNAs, tend to correlate well with increased *de novo* transcription in flies [20] and mice [33]. We performed *in organello* transcription assays in larvae at 3–5 days ael and found no clear difference at 3 days ael, whereas there was an increase of *de novo* transcription of mtDNA in KD larvae at 4 and 5 days ael (Figure 5A).

### Loss of *DmMterf3* leads to disturbed mitochondrial ribosome assembly and impaired mitochondrial translation

The combination of a respiratory chain deficiency (Figure 3) and transcription activation (Figure 4C–4F and Figure 5A) suggested that either the activation of *de novo* transcription leads to a respiratory chain deficiency, e.g. by causing the observed imbalance in steady-state levels of transcripts, as previously suggested for the *Mterf3* knockout mouse [5], or, alternatively, that the transcriptional activation is a secondary response to respiratory chain deficiency. The observation of decreased 16S rRNA levels (Figure 4C, 4E and Figure S5B, S5C) were interesting in this respect because the *Mterf3* knockout mouse also displays such a decrease [5]. We therefore continued with a more detailed characterization of mitochondrial translation in the *DmMterf3* KD larvae (Figure 5B–5D).

First, we determined the assembly states of the mitochondrial ribosomes by sedimentation gradient centrifugation of mitochondrial extracts isolated from larvae at 3 and 5 days ael (Figure 5B, 5C and Figure S6A). Fractions were collected across the gradient and analyzed for absorption at 260 nm to determine the RNA content in each fraction. Equal loading was assessed by analyzing an aliquot of the total protein extract, to be loaded on the gradient, on a SDS-PAGE gel followed by Coomassie staining (Figure S6B). Fractions were thereafter analyzed by qRT-PCR to measure levels of 12S and 16S rRNA. Control samples confirmed that we indeed were able to separate the small (28S) subunit, the large (39S) subunit and the assembled (55S) ribosome (Figure 5B, 5C). Already in *DmMterf3* KD larvae at 3 days ael, we observed a reduction in levels of the assembled 55S ribosome (Figure 5B). This decrease of assembled ribosomes was even more pronounced in *DmMterf3* KD larvae at 5 days ael and was, at this time point, accompanied by a marked increase of the 28S ribosomal subunit and a marked decrease of the 39S ribosomal subunit (Figure 5C).

To further study the consequences of reduced levels of the assembled ribosome, we performed assays to determine the *de novo* translation activity in isolated mitochondria and found a clear decrease in *DmMterf3* KD larvae at 3 days ael and onwards (Figure 5D). Our results suggest that the reduced mitochondrial translation is caused by a problem with ribosome assembly and that the concomitant transcriptional response with imbalanced steady-state transcript levels may be a contributing factor, thus suggesting a link between these processes.

### Loss of MTERF3 in the mouse also leads to decreased levels of the assembled large ribosomal subunit

The suggestion that DmMTERF3 might play a direct role in mitochondrial ribosome biogenesis prompted us to re-investigate the *Mterf3* knockout mice. We previously created *Mterf3* heart knockout mice by crossing *Mterf3^loxP^* mice to transgenic mice expressing *cre*-recombinase under the control of the muscle creatine kinase promoter (*Ckmm-cre*) [5]. Deletion of MTERF3 in the heart leads to a severe respiratory chain deficiency, progressive increase in steady-state levels of most mitochondrial transcripts and profound increase of *de novo* mtDNA transcription [5]. In the knockout hearts, MTERF3 protein levels are severely reduced already at 4 weeks of age (Figure S7A), concomitant with a dramatic increase of *de novo* transcription (Figure S7B and [5]).

**Figure 6:**
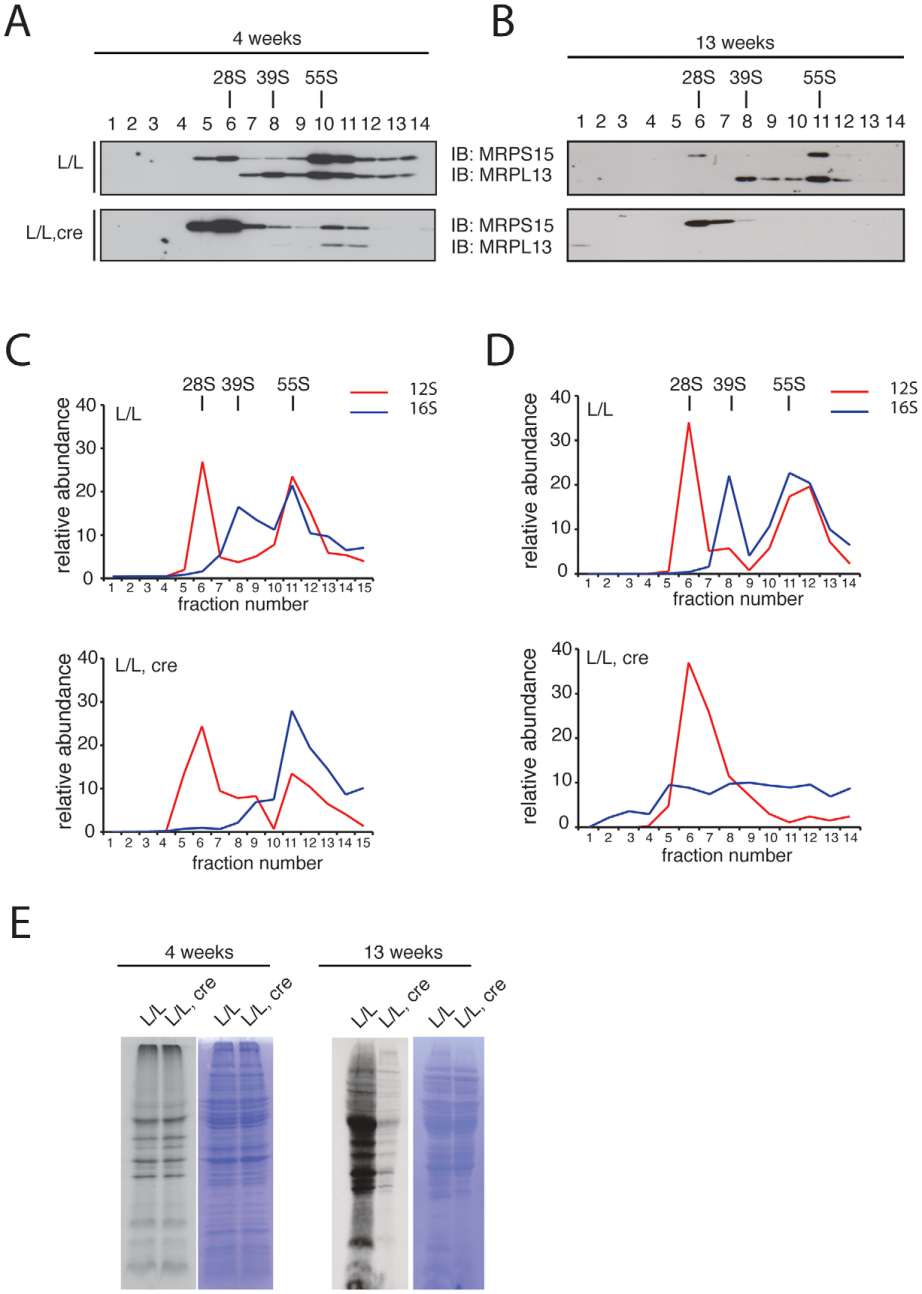
Mitochondrial ribosome assembly and *de novo* translation in hearts of *Mterf3* knockout mice. (A) Sedimentation analysis of ribosomal assembly in isolated mitochondria from 4-week-old control (L/L) and *Mterf3* knockout (L/L, *cre*) mouse hearts. Individual fractions were separated by SDS-PAGE, followed by Western blotting using antibodies against protein components of the small (MRPS15) and large (MRPL13) ribosomal subunit. The small (28S) ribosomal subunit, the large (39S) ribosomal subunit and the assembled (55S) ribosome are indicated. (B) Sedimentation analysis of ribosome assembly in isolated mitochondria from 13-week-old control (L/L) and *Mterf3* knockout (L/L, *cre*) mouse hearts analyzed as in (A). (C) The individual mitochondrial rRNAs in fractions from (A) were detected using TaqMan-specific probes. The abundance of rRNA in each fraction is shown as percentage from the sum of the abundance in all fractions. (D) The individual mitochondrial rRNAs in fractions from (B) were detected using TaqMan-specific probes as in (C). (E) Analysis of mitochondrial *de novo* translation as determined by *in organello* S^35^-methionine incorporation into newly synthesized proteins in mitochondria from control (L/L) and *Mterf3* knockout (L/L, *cre*) hearts at age 4 and 13 weeks. The total mitochondrial protein content, as well as the coomassie stainings presented next to the autoradiogramms, were used to monitor loading of the gels.

We proceeded to investigate the assembly of the mitochondrial ribosomal subunits in *Mterf3* knockout mouse heart mitochondria, by gradient sedimentation and Western blot analysis. The 28S and 39S ribosomal subunits, as well as the fully assembled 55S ribosome were clearly resolved in control samples, as determined by the migration of the ribosomal subunit markers MRPS15 and MRPL13 (Figure 6A, 6B). In contrast, in *Mterf3* heart knockout samples the amount of MRPL13 co-migrating with MRPS15 was severely reduced already at 4 weeks of age, suggesting a reduction of fully assembled ribosomes (Figure 6A). Concomitant with the reduction of 55S ribosomes, we observed increased levels of the free 28S ribosomal subunit (Figure 6A). The MRPL13 protein steady-state levels progressively decreased (Figure S7C) and by 13 weeks of age, no fully assembled ribosomes were detectable (Figure 6B). These results clearly suggest that the assembly of the mitochondrial ribosome is impaired in the absence of MTERF3. We performed a set of confirmatory experiments, where we used qRT-PCR to assess presence of 12S and 16S rRNA in the different fractions. As predicted, the relative levels of 16S rRNA were reduced in the fraction corresponding to the 39S ribosomal subunit already at 4 weeks of age in *Mterf3* heart knockout mitochondria (Figure 6C). This reduction became even more pronounced at later stages and there was eventually a complete loss of the fully assembled ribosomes (Figure 6D). The partial co-migration of MTERF3 and MRPL13 on sucrose gradients (Figure S7D) suggests that MTERF3 is involved in the maturation of the 39S subunit. Next, we investigated whether the impaired ribosomal assembly affected mitochondrial translation by performing *in organello de novo* translation experiments, which showed no change at the age of 4 weeks and severely decreased translation in 13-week-old *Mterf3* knockout heart mitochondria (Figure 6E). A similar global decrease in mitochondrial translation has also been reported in *Mterf3* knockdown *Drosophila* cell lines [38].

### MTERF3 specifically binds to mitochondrial 16S ribosomal RNA

**Figure 7:**
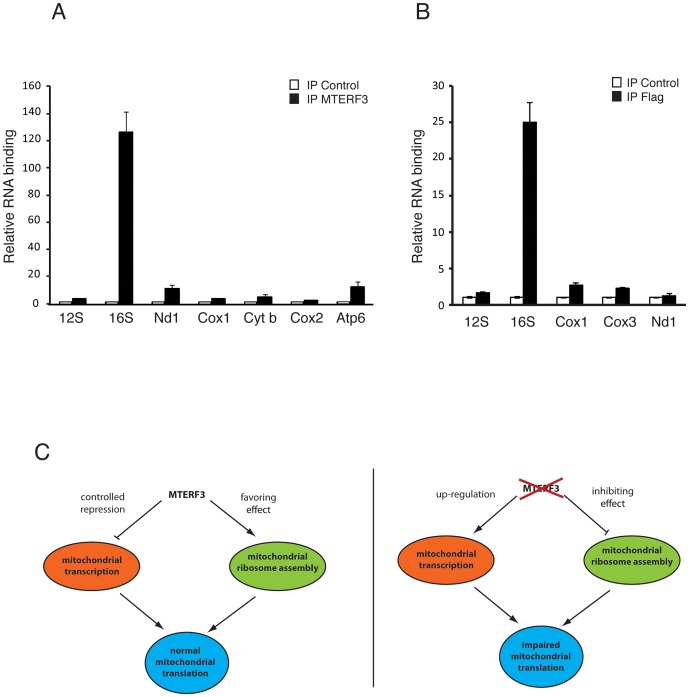
Binding of MTERF3 to mitochondrial 16S rRNA and model of MTERF3 action. (A) Identification of mitochondrial transcripts interacting with MTERF3 in mice was performed by co-immuno-precipitation using a mouse anti-MTERF3 antibody. Transcripts bound to mouse MTERF3 were quantified using qRT-PCR and their abundance is shown as percentage of levels in control IPs. (B) Identification of mitochondrial transcripts interacting with MTERF3 in flies was performed by co-immuno-precipitation using an anti-FLAG antibody and extracts from flies expressing DmMTERF3-linker-Flag. Transcripts bound to fly MTERF3 were quantified using qRT-PCR and their abundance is shown as percentage of levels in control IPs. Error bars correspond to standard deviation. (C) Model of the physiological role of MTERF3 in control of mtDNA expression (left panel) and model of the phenotypes caused by loss of MTERF3 (right panel).

We observed that loss of MTERF3 leads to reduced 39S ribosomal subunit assembly and a concomitant decrease of levels of the fully assembled 55S ribosome, in both flies and mice. These results are somewhat reminiscent of the findings in *Mterf4* heart knockouts [33], which show accumulation of apparently normal 28S and 39S ribosomal subunits, but a severe reduction in levels of the fully assembled 55S ribosome. Loss of MTERF3 is associated with a drastic reduction of 16S rRNA and impaired assembly of the 39S large ribosomal subunit, suggesting that the 16S rRNA may be interacting with MTERF3. We therefore performed electrophoretic gel mobility shift assays (EMSA) by incubating constant amounts of DNA and RNA templates with increasing amounts of recombinant human MTERF3 protein. Non-specific double- (ds) and single-stranded (ss) DNA or RNA templates with an identical 28 bases long arbitrary sequence only interacted weakly with MTERF3 (Figure S8A, S8B), whereas mitochondrial ribosomal RNA templates showed a stronger binding (Figure S8C, S8D). These binding assays support the prediction that MTERF3 preferentially binds mitochondrial ribosomal RNA. To further characterize MTERF3 interactions with mitochondrial rRNA *in vivo*, we performed RNA-immuno-precipitation (RNA-IP) in mitochondrial preparations from wild-type mouse heart and fly larvae. The lack of a suitable DmMTERF3 antibody prompted us to generate a transgenic fly line expressing a Flag-tagged form of DmMTERF3 under the inducible UAS-GAL4 system. Expressing DmMTERF3 with a Flag tag directly at the C-terminus leads to an unstable protein not detectable by Western blotting. We therefore introduced a linker sequence between the C-terminus of DmMTERF3 and the Flag tag, and confirmed the expression of this tagged protein by Western blot (Figure S9A). Control experiments demonstrated that we were able to efficiently immuno-precipitate endogenous mouse MTERF3 or Flag-tagged DmMTERF3 proteins (Figure S9B, S9C). RNA-IP clearly demonstrated a very specific interaction between MTERF3 and 16S rRNA both in mouse and fly samples (Figure 7A, 7B).

## Discussion

Regulation of mtDNA expression is important to fine-tune oxidative phosphorylation in response to physiological demand and pathological states. This regulation may occur at many different levels and mitochondria are in essence a prokaryotic system where transcription and translation occur within the same compartment, the mitochondrial matrix, and therefore likely directly interact in a molecular crosstalk. The roles of many of the involved factors are poorly understood. The 39S ribosomal subunit MRPL12 and the posttranscriptional regulator LRPPRC have both been implicated in activation of transcription [18], [39], but these proposed roles are not supported by other studies [14], [19]. MTERF3 has previously been identified as a negative regulator of mtDNA transcription in mammals [5], but its molecular mode of action has remained difficult to assess. Based on the knowledge that the mtDNA gene content is conserved among metazoans, we hypothesized that key regulatory processes controlling mtDNA expression also should be conserved. We therefore decided to take a novel approach to investigate MTERF3 function by creating knockout and knockdown fruit flies with abolished or reduced DmMTERF3 expression. Similar to the mouse, we found that loss of DmMTERF3 results in lethality and activation of mtDNA transcription. Unexpectedly, we could also identify a novel role for DmMTERF3 in mitochondrial ribosome assembly by regulation of the biogenesis of the 39S ribosomal subunit. The biogenesis of the 28S ribosomal subunit was not affected in the absence of DmMTERF3 and instead this subunit accumulated as it could not be assembled into a functional ribosome in the absence of the 39S subunit. Reinvestigation of the *Mterf3* knockout phenotype in the mouse showed a similar assembly defect of the 39S subunit, which was present already in early stage knockout animals. In summary, MTERF3 has a novel function in regulation of ribosomal biogenesis and loss of MTERF3 expression does not only impair translation but also causes activation of mtDNA transcription in both flies and mice.

The crystal structure of MTERF3 has been solved at 1.6 Å resolution [40] and predicts that the protein binds nucleic acids. The crystal structure of MTERF1 bound to mtDNA has given novel mechanistic insights how such a binding can occur [28], [29]. On chromatin immuno-precipitation analysis, MTERF3 binds the promoter region of mammalian mtDNA and depletion of MTERF3 from a human mitochondrial extract leads to activation of mtDNA transcription [5]. These effects of MTERF3 on transcription may be due to direct interaction with mtDNA, but it is also possible that MTERF3 modulates transcription by binding the nascent RNA emerging after transcription initiation. In this report, we performed a series of gel-shift analyses, which show that MTERF3 displays weak binding to single- or double-stranded RNA or DNA of random sequence. However, recombinant MTERF3 has a marked preference for binding mitochondrial rRNA fragments, containing both single and double stranded regions. RNA-IP studies further demonstrated a strong and specific interaction of MTERF3 with the 16S rRNA *in vivo*, suggesting that MTERF3 contributes to 16S rRNA stabilization and/or modification and thereby explaining the critical role for MTERF3 in the biogenesis of the 39S ribosomal subunit. We propose that without this putative 16S rRNA modification, the 39S ribosomal subunit cannot be properly assembled, which, in turn, leads to a severe translational defect. There is strong precedence that abolished modification of mitochondrial rRNAs can affect the assembly of the ribosome. The best understood example is TFB1M, which is an adenine methyltransferase that dimethylates two highly conserved adenines at a stem loop structure close to the 3′ end of 12S rRNA in mammalian mitochondria [23]. Another well characterized example is MTERF4, which interacts with NSUN4 and brings this cytosine methyltransferase to the large ribosomal subunit, where it is thought to modify 16S rRNA [33], [34].

In bacteria, transcription and translation are coordinated, and the rate of transcription is tightly coupled to the processivity of the translating ribosome [41]. Mitochondria may coordinate gene expression in a similar way, where transcription and translation are oppositely coordinated, because loss of assembled ribosomes leads to a massive increase in *de novo* transcription. Interestingly, knockout of *Mterf3* [5], *Tfb1m* [23] and *Mterf4* [33] all cause a severely defective translation and a dramatic increase in *de novo* transcription, with increased steady-state levels of most or all mitochondrial transcripts. These findings suggest that one of the early responses to a ribosomal assembly defect is a massive transcriptional activation. It is interesting to note that the MTERF3 protein levels are down-regulated in both *Tfb1m* [23] and *Mterf4* [33] knockouts, suggesting further that MTERF3 may have a key role in mediating the effects on transcription, and that up-regulation of mitochondrial transcription in the absence of MTERF3 cannot simply be attributed to a passive compensatory mechanism. We propose that MTERF3 promotes translation by regulation of ribosomal biogenesis and that this process is linked to repression of mtDNA transcription activation (Figure 7C). In the absence of MTERF3, the ribosomal biogenesis is impaired and there is an increased uncontrolled activation of mtDNA transcription leading to imbalanced steady-state levels of mitochondrial transcripts (Figure 7C). Our present data in combination with previous reports [5] suggest that MTERF3 could have a dual function and be a part of a molecular checkpoint, acting to coordinate transcriptional and translational rates and thereby optimizing mtDNA expression.

Unraveling the function of specific proteins is not always easy in mammalian systems and many of the methods used to study protein functions and interactions are plagued by experimental ambiguities. Here, we describe a strategy that takes advantage of genetic manipulation of the orthologous gene in two distantly related metazoans, accompanied by a comprehensive molecular characterization. By using this strategy, we present compelling evidence that MTERF3 has a conserved role in ribosomal biogenesis in metazoans and that it also coordinates mitochondrial transcription and translation. At least two members of the mammalian MTERF family, i.e. MTERF3 and MTERF4, have now been found to have critical roles in mitochondrial ribosomal biogenesis. This makes it tempting to speculate that MTERF1 and MTERF2 could have similar, yet undiscovered, roles in ribosomal biogenesis. Future studies will have to clarify whether MTERF3 has protein interaction partners that are involved in modifying rRNA or if MTERF3 is essential for ribosomal biogenesis by some other mechanism.

## Materials and Methods

### Ethics statement

This study was performed in strict accordance with the recommendations and guidelines of the Federation of European Laboratory Animal Science Associations (FELASA). The protocol was approved by the “Landesamt für Natur, Umwelt und Verbraucherschutz Nordrhein-Westfalen”.

### Rescue experiments

In order to rescue phenotypes caused by *DmMterf3* RNAi expression, we co-expressed the *Mterf3* gene from *Drosophila pseudoobscura* (Dp), which is not a target of the *DmMterf3* RNAi line [42]. The fosmid clone FlyFos047383 that contains the *DpMterf3* gene was kindly provided by Dr. Pavel Tomancak (MPI for Cell Biology, Dresden, Germany). A 10 kb Fosmid fragment containing the *DpMterf3* gene was cloned into the pBluescript II Sk+ vector (Stratagene) by ET recombination. Subsequently, the 10 kb fragment was released by *Not*I and *Bgl*II restriction enzyme cleavage and subcloned into the transfection vector pattB [43]. Oligonucleotide primers used for cloning are listed in Table S1. Embryo injections to achieve site-specific integration into attP40 flies were performed by Best Gene Drosophila Embryo Injection Services (Chino Hills, California, USA).

### Generation of *DmMterf3* mutants by ends-out homologous recombination

*DmMterf3* null mutants were generated by ends-out homologous recombination as described [37]. Approximately 4 kb of 5′ and 3′ flanking sequences of the *DmMterf3* gene were cloned into the pBluescript II Sk+ vector (Stratagene) by ET recombination, using a *DmMterf3* BAC clone as template (BACPAC Resource Center, Oakland, California, USA). Both 5′ and 3′ homologous arms were sequenced to ensure the absence of base substitutions and subsequently subcloned into the pGX attP vector [44] to generate the *DmMterf3* targeting plasmid. Primer sequences and restriction sites used for subcloning into the pGX attP vector are listed in Table S1. The targeting plasmid was injected into *D. melanogaster* embryos via P-element-mediated germ line transformation using the Best Gene Drosophila Embryo Injection Services (Chino Hills, California, USA). Crosses for ends-out homologous recombination were carried out as described [37]. Homologous recombination events were identified by PCR. Subsequently, the white (hs) marker was removed using *cre*-recombinase and the absence of the *DmMterf3* gene was confirmed by PCR and sequencing (primers are listed in Table S1). The maintenance of the fly lines is described in Text S1.

### Generation of transgenic flies expressing Flag tagged *DmMterf3*

The *DmMterf3* cDNA clone LD27042 was purchased form DGRC and subsequently cloned into the transfection vector pUASTattB. A Flag tag was linked to the C-terminus of DmMTERF3 via a linker sequence (GAAAAGAAAAG), generating the DmMTERF3-linker-Flag construct. Oligonucleotide primers used for cloning are listed in Table S1. The construct was embryo injected into attP40 flies for generation of the transgenic flies.

### Respiratory rates

Fly larvae (n = 3–7) were dissected in PBS and resuspended in 2 ml of respiratory buffer (120 mM sucrose, 50 mM KCl, 20 mM Tris-HCl, 4 mM KH2PO4, 2 mM MgCl2, 1 mM EGTA, 0.01% digitonin, pH 7.2). Oxygen consumption was measured at 25°C using an oxygraph chamber (OROBOROS). Complex I-dependent respiration was assessed by adding the substrates proline (10 mM), pyruvate (10 mM), malate (5 mM) and glutamate (5 mM). Succinate and glycerol-3-phosphate dehydrogenase activities were measured using 20 mM succinate (SUCC) and 15 mM glycerol-3-phosphate (G3P), respectively. The mitochondrial quality of each sample was assessed by measuring the respiratory control rate (RCR) using 1 mM ADP (state 3) or 1 mM ADP and 2.5 µg/ml oligomycin (pseudo state 4). Permeabilized control mitochondria consistently had RCR values between 4 and 7 with complex I substrates. The respiration was uncoupled by the addition of 400 µM CCCP and the rotenone-sensitive flux was measured in the presence of 200 µM rotenone. Finally, the protein content was determined by the Bradford method (BioRad) in order to normalize the oxygen consumption flux to mitochondrial protein content.

### *In organello* transcription and translation assays

Mitochondria were isolated from fly larvae or mouse hearts and *in organello* transcription assays were performed as described [45] by incubating 200 µg mitochondria in a modified transcription buffer (30 µCi [α-^32^P]-UTP, 25 mM sucrose, 75 mM sorbitol, 100 mM KCl, 10 mM K2HPO4, 50 µM EDTA, 5 mM MgCl2, 1 mM ADP, 10 mM glutamate, 2.5 mM malate, 10 mM Tris-HCl (pH 7.4) and 5% (w/v) BSA) for 45 min. Labeled mitochondrial RNA was isolated using Totally RNA kit (Ambion), separated on a 1.2% agarose gel and blotted to Hybond-N+ membranes (GE Healthcare).

*In vitro* assays to study mitochondrial *de novo* translation with [^35^S]-methionine were performed as described [20] and equal amounts of total mitochondrial protein were separated on 15% SDS-PAGE gels. Gels were fixed in isopropanol-acetic solution, stained with Coomassie, destained in ethanol-acetic acid solution and treated with Amplify Solution (GE Healthcare). Afterwards gels were dried and [^35^S]–methionine-labeled proteins were visualized by autoradiography. For *in organello* transcription and translation fly mitochondria were incubated at 30°C and mouse heart mitochondria at 37°C.

### Sucrose density gradient analysis of 28S and 39S ribosomal subunits

Mitochondrial ribosomes from fly larvae or mouse hearts were prepared as previously described [23], [46]. Mitochondrial ribosomes were loaded onto 10–30% sucrose gradients and separated by centrifugation overnight. From sucrose gradients, fractions (∼500 µl) were collected with continuous monitoring of absorbance at 260 nm. RNA was extracted from each fraction using TRIzol LS Reagent (Invitrogen) according to manufacturer's recommendations, subsequently treated with DNase and used for cDNA synthesis. Absolute qRT-PCR analysis using a standard curve composed of an equal amount of RNA from each fraction from the control and the KD group, was performed using SYBR green master mix and primers specific for 12S and 16S rRNA, as described in Text S1.

Fractions (750 µl) were collected from the mouse heart sucrose gradients and proteins in each fraction were precipitated with trichloracetic acid and subjected to SDS-PAGE followed by immunoblotting. Sub-ribosomal particles were detected using antisera specific for individual proteins from the 28S and 39S ribosomal subunits, as described in Text S1. RNA was extracted from each fraction using TRIzol LS Reagent (Invitrogen) according to manufacturer's recommendations, subsequently treated with DNase and used for cDNA synthesis and qRT-PCR with TaqMan probes specific for 12S and 16S rRNA [19].

### RNA immunoprecipitation

Mitochondria were isolated by differential centrifugation from control (w;;da-GAL4/+) and MTERF3-linker-Flag expressing (w;;UAS-DmMterf3:linker:Flag/+;da-GAL4/+) larvae and from wild-type mouse heart mitochondria. RNA-IP was performed essentially as previously described [33]. The final mitochondrial pellet was suspended in a low-salt NET-2 lysis buffer buffer (50 mM Tris-HCl [pH 7.4], 150 mM NaCl, 0.05% Nonidet P-40, 1× complete EDTA-free protease inhibitor cocktail [Roche]) supplemented with 100 U of RNasin Plus (Promega). After 20 min incubation on ice, the mitochondrial lysates were centrifuged at 10,000 g for 10 min at 4°C in order to pellet the debris. Supernatants were collected and protein concentrations determined by Bradford-based assay (Sigma). IPs were performed using ∼200 µg mitochondrial protein, and lysates were pre-cleared with agarose beads (Sigma) by rotation for 1 h at 4°C, followed by a 2 h incubation at 4°C with Anti-Flag M2 Affinity Gel (Sigma) or a mix of protein A agarose/protein G agarose (Roche) coupled to a polyclonal antibody directed against mouse MTERF3 (peptide Specialty Laboratory), for fly or mouse samples, respectively. Both, anti-Flag M2 Affinity gel and protein A/protein G agarose bead mix coupled to MTERF3 antibody, were incubated for 1 h with 100 µg yeast tRNA prior to usage. After incubation with fly or mouse mitochondria, beads were washed by rotation for 2×10 min at 4°C in low-salt NET-2 buffer, followed by 2×5 min washes in high-salt NET-2 buffer (50 mM Tris-HCl [pH 7.4], 300 mM NaCl, 0.05% Nonidet P-40, 1× complete EDTA-free protease inhibitor cocktail [Roche]), and a final wash for 4×10 min in low-salt NET-2 buffer. The washed beads were resuspended in 120 µl reversion buffer (50 mM Tris-HCl [pH 6.8], 1% SDS, 5 mM EDTA, 10 mM DTT) supplemented with RNasin Plus (Promega) and incubated for 45 min at 65°C. RNA was recovered by TRIzol extraction (Invitrogen) following manufacturer's recommendations, using 10 µg yeast tRNA (Ambion) as a carrier. RNA was subjected to DNase treatment (Turbo DNA-free kit, Ambion) and reverse transcribed to cDNA by using the High-Capacity cDNA Archive kit (ABI). Mitochondrial transcripts from the RNA-IP experiments were identified and quantified by qRT-PCR, with non-primed beads used as background controls.

## Supporting Information

Figure S1 *DmMTERF3* encodes a conserved mitochondrial protein. (A) Phylogenetic tree of the MTERF family of proteins. (B) S2R+ (upper panel) and HeLa cells (lower panel) expressing a GFP-tagged DmMTERF3 fusion protein (DmMterf3-FLAG-GFP) counterstained with Mitotracker Deep Red. The scale bar size is 10 µm.
(TIF)

Figure S2 Phenotype and molecular characterization of tissue-specific *DmMterf3* knockdown flies. (A) Eye-specific knockdown of *DmMterf3* showing reduced eye size. (B) Disorganized head structures in flies with eye-specific knockdown of *DmMterf3.*
(TIF)

Figure S3 Similarity between *DmMterf3* and *DpMterf3*. (A) Alignment of the nucleotide sequences for *DmMterf3* and *DpMterf3*. Identical nucleotides are marked in black. (B) Alignment of the amino acid sequences for DmMTERF3 and DpMTERF3. Identical amino acids are marked in black.
(TIF)

Figure S4 DmMTERF3 is important for respiratory chain function. (A) Western blot analysis using antibodies against the nuclear-encoded NDUFS3 subunit of complex I and the nuclear-encoded α-subunit of ATP synthase (complex V) in control and *DmMterf3* knockout larvae at 3 days ael. Porin was used as a loading control. (B) Coomassie staining to assess loading of BN-PAGE gels used to assess in-gel activity of complex I and IV in mitochondrial protein extracts from control and *DmMterf3* KD larvae at 6 days ael.
(TIF)

Figure S5 MtDNA and tRNA levels in *DmMterf3* knockdown larvae. (A) Q-PCR analysis of mtDNA levels in control and *DmMterf3* KD larvae 5 and 6 days ael. (B) Northern blot analysis of steady-state levels of mitochondrial large ribosomal RNA (16S rRNA) in control and *DmMterf3* KD larvae at 3 days ael. Loading of gels was normalized to the transcript encoding the nuclear ribosomal protein 49. (C) Quantification of Northern blot analysis to assess steady-state levels of mitochondrial 16S rRNA in control and *DmMterf3* KD larvae at 3 days ael. Loading of gels was normalized to the transcript encoding the nuclear ribosomal protein 49. (D) Northern blot analysis of steady-state levels of mitochondrial tRNAs in control and *DmMterf3* KD larvae at 5 days ael. Loading of gels was normalized to nuclear 5S ribosomal RNA. (E) Quantification of Northern blot analysis to assess steady-state levels of mitochondrial tRNAs in control and *DmMterf3* KD larvae at 5 days ael. Loading of gels was normalized to nuclear 5S ribosomal RNA.
(TIF)

Figure S6 Sedimentation analysis of ribosomes in control and *DmMterf3* knockdown flies. (A) Sedimentation analysis of ribosomal assembly in control and *DmMterf3*KD larvae at 3 days ael. The small (28S) ribosomal subunit, the large (39S) ribosomal subunit and the assembled (55S) ribosome were identified by the increased absorbance at 260 nm as indicated. (B) Aliquots of the mitochondrial protein extracts analyzed in (A) were separated by SDS-PAGE followed by Western blot analysis, using PVDF membranes, that were stained with Coomassie to assess loading.
(TIF)

Figure S7 MTERF3 protein levels, *de novo* transcription and steady-state level of mitochondrial ribosomal proteins in control and *Mterf3* knockout mouse hearts at 4 and 13 weeks of age. (A) Western blot analysis using an antibody against mouse MTERF3. VDAC is used as a loading control. (B) *In organello* transcription assays were performed in isolated crude mitochondrial preparations from control (L/L) and *Mterf3* knockout (L/L, *cre*) mouse hearts at 4 and 13 weeks of age. VDAC is used as a loading control. (C) Western blot analysis of MRPL13 and MRPS15 steady-state protein levels in mitochondrial extracts from control (L/L) and *Mterf3* knockout (L/L, cre) hearts at different ages. VDAC is used as a loading control. (D) Sedimentation analysis of the 28S, 39S, 55S and MTERF3 in wild-type heart mitochondria from 13-week-old mice, by centrifugation through a linear 10%–30% density sucrose gradient. The different fractions (numbered) were analyzed by SDS-PAGE and subsequent Western-blotting.
(TIF)

Figure S8 Binding of MTERF3 to DNA and RNA templates. (A) Binding of MTERF3 to double-stranded (ds) non-specific DNA or RNA. (B) Binding of MTERF3 to single-stranded (ss) non-specific DNA or RNA. (C) Binding of MTERF3 to mitochondrial 16S rRNA. (D) Binding of MTERF3 to mitochondrial 12S rRNA templates. The assays in (A–D) were performed with the following MTERF3 protein concentrations: 0, 0.02, 0.04, 0.08, 0.16, 0.36, 0.64, 1.28, 2.56 mM. The template amount was 40 ng for the experiments shown in (A–D). Free and bound templates are indicated.
(TIF)

Figure S9 Stable expression of FLAG tagged DmMterf3 and MTERF3 immuno-precipitations. (A) Western blot analysis using an anti-Flag antibody (IB: Flag) on mitochondrial extracts from transgenic fly lines expressing DmMTERF3-linker-Flag. Porin is used as a loading control (IB: Porin). (B) DmMTERF3 was immuno-precipitated from Dm-MTERF3-linker-Flag-expressing flies using an anti-Flag resine (IP: Flag) and the same conditions as those used for RNA-IP. The different fractions were then blotted with an anti-Flag antibody (IB: Flag). (C) MTERF3 was immuno-precipitated (IP: MTERF3) from wild-type mouse heart mitochondria with the same conditions as those used for the RNA-IP. The different fractions were blotted with a monoclonal antibody directed against MTERF3 (IB:MTERF3).
(TIF)

Table S1 List of the primers used for cloning and qRT–PCR experiments.
(DOC)

Text S1 Supporting materials and methods including supporting references.
(DOC)
