# Supplementary figures and images for: MTERF3 Regulates Mitochondrial Ribosome Biogenesis in Invertebrates and Mammals
Source: PLoS Genet. 2013 Jan 3;9(1):e1003178. doi: 10.1371/journal.pgen.1003178 (PMC3536695; doi:10.1371/journal.pgen.1003178)

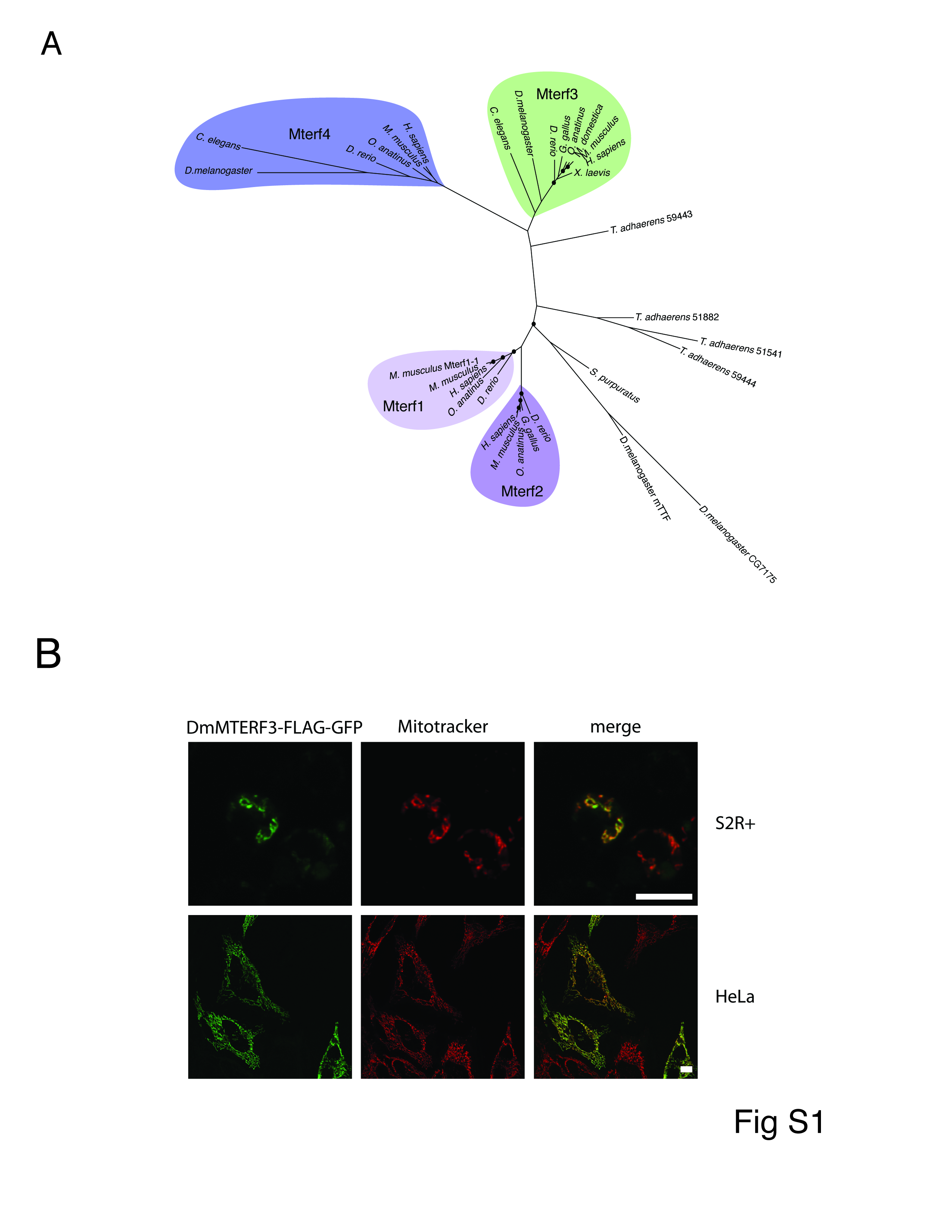

Supplement: Figure S1 — DmMTERF3 encodes a conserved mitochondrial protein. (A) Phylogenetic tree of the MTERF family of proteins. (B) S2R+ (upper panel) and HeLa cells (lower panel) expressing a GFP-tagged DmMTERF3 fusion protein (DmMterf3-FLAG-GFP) counterstained with Mitotracker Deep Red. The scale bar size is 10 µm. (TIF) [file pgen.1003178.s001.tif]

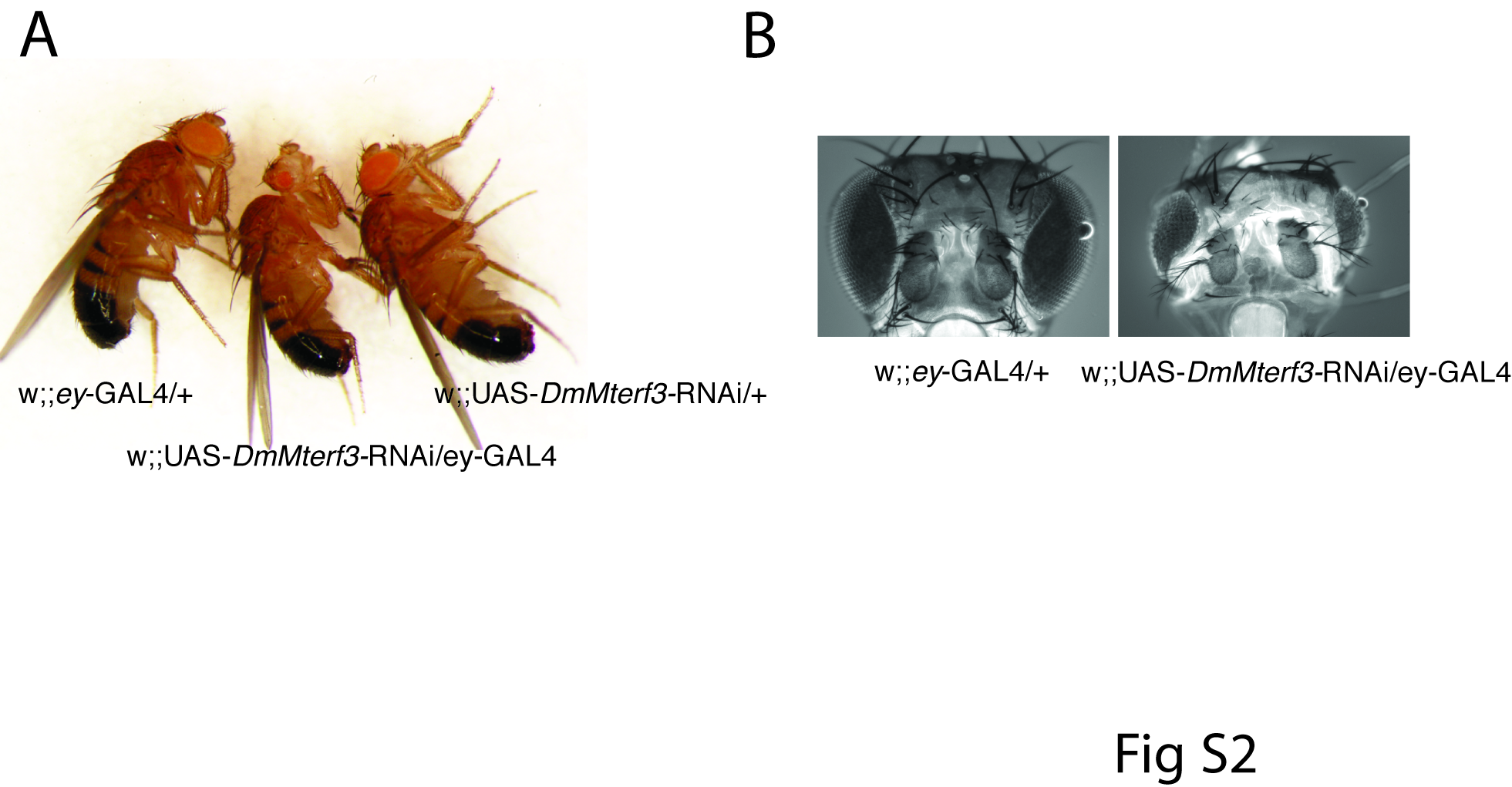

Supplement: Figure S2 — Phenotype and molecular characterization of tissue-specific DmMterf3 knockdown flies. (A) Eye-specific knockdown of DmMterf3 showing reduced eye size. (B) Disorganized head structures in flies with eye-specific knockdown of DmMterf3. (TIF) [file pgen.1003178.s002.tif]

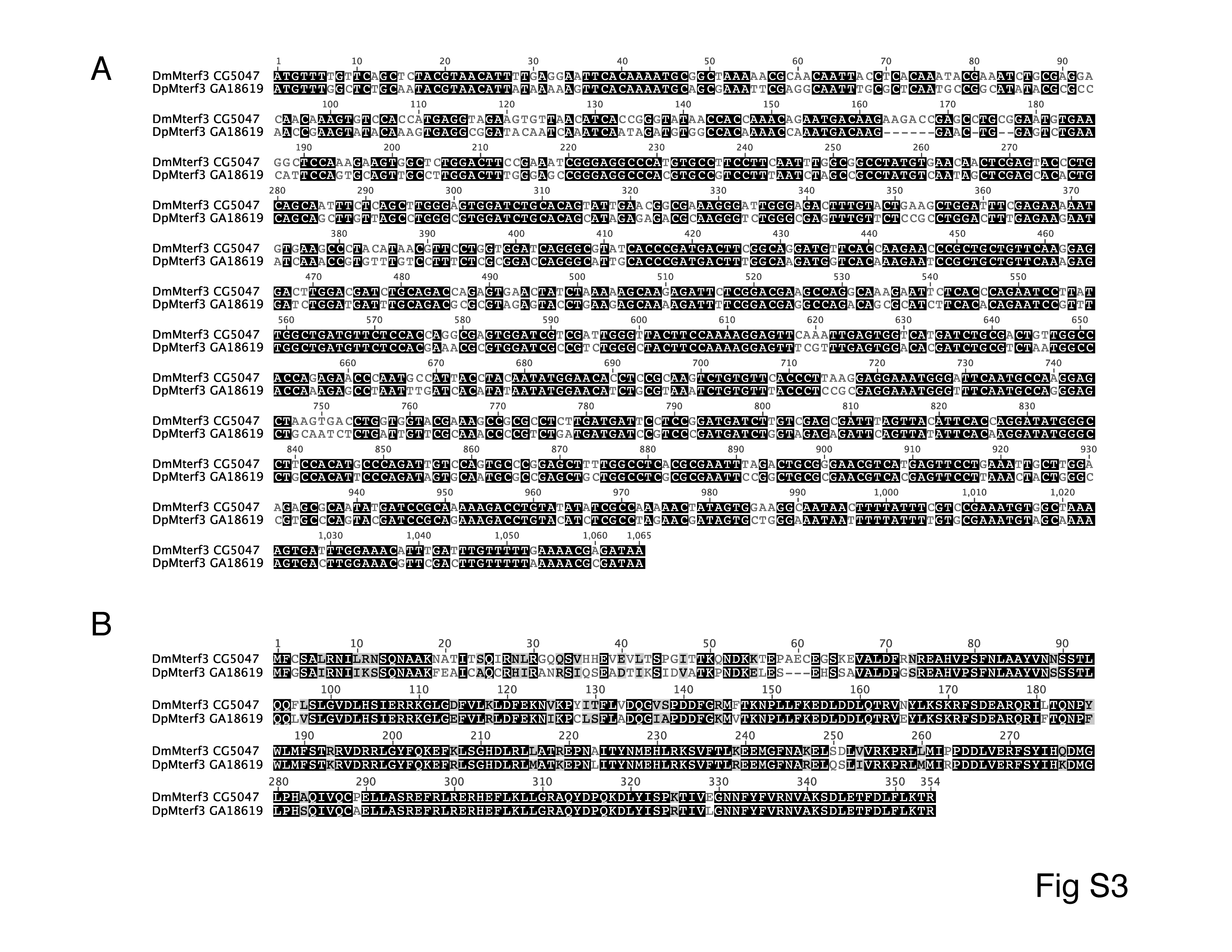

Supplement: Figure S3 — Similarity between DmMterf3 and DpMterf3. (A) Alignment of the nucleotide sequences for DmMterf3 and DpMterf3. Identical nucleotides are marked in black. (B) Alignment of the amino acid sequences for DmMTERF3 and DpMTERF3. Identical amino acids are marked in black. (TIF) [file pgen.1003178.s003.tif]

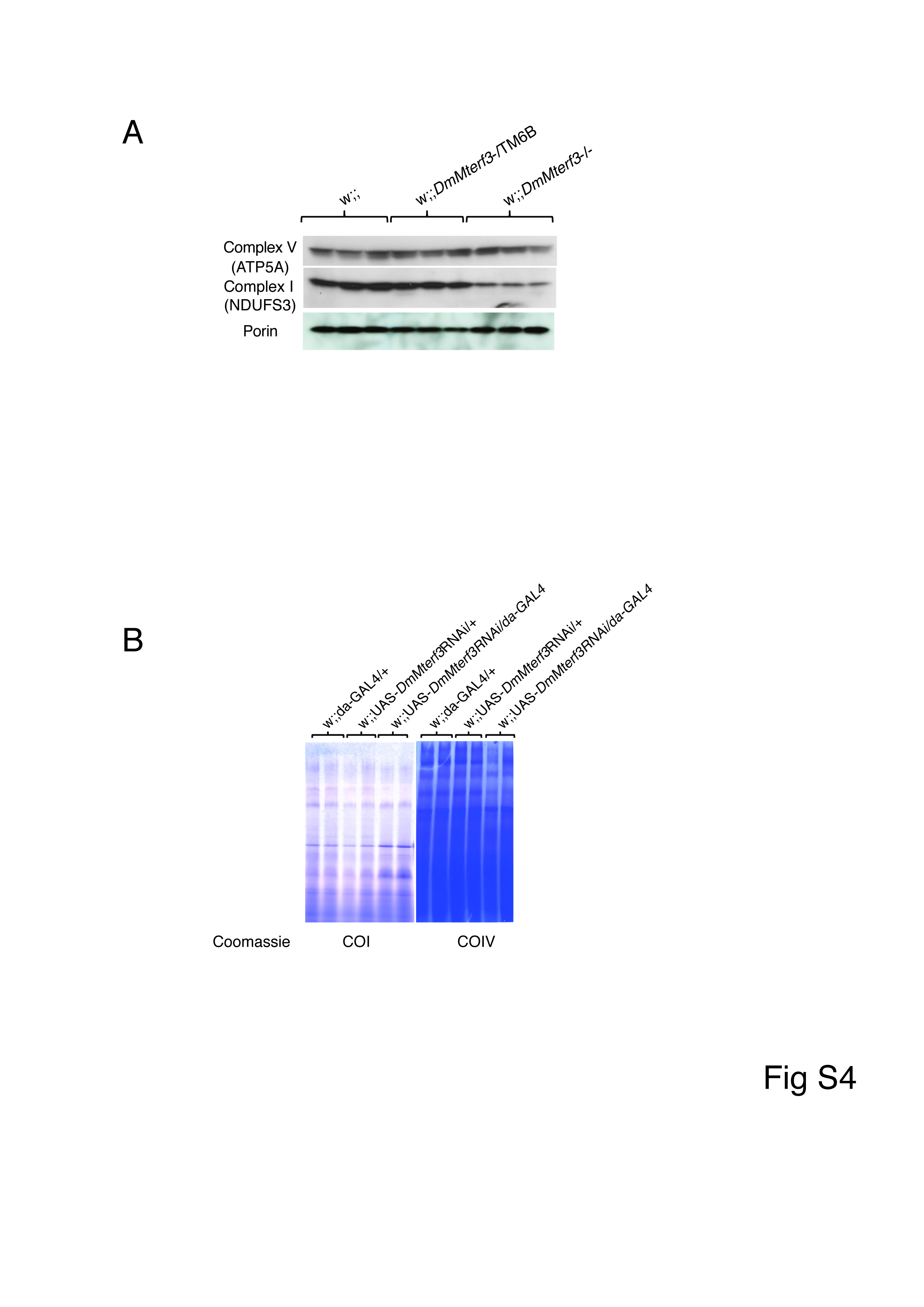

Supplement: Figure S4 — DmMTERF3 is important for respiratory chain function. (A) Western blot analysis using antibodies against the nuclear-encoded NDUFS3 subunit of complex I and the nuclear-encoded α-subunit of ATP synthase (complex V) in control and DmMterf3 knockout larvae at 3 days ael. Porin was used as a loading control. (B) Coomassie staining to assess loading of BN-PAGE gels used to assess in-gel activity of complex I and IV in mitochondrial protein extracts from control and DmMterf3 KD larvae at 6 days ael. (TIF) [file pgen.1003178.s004.tif]

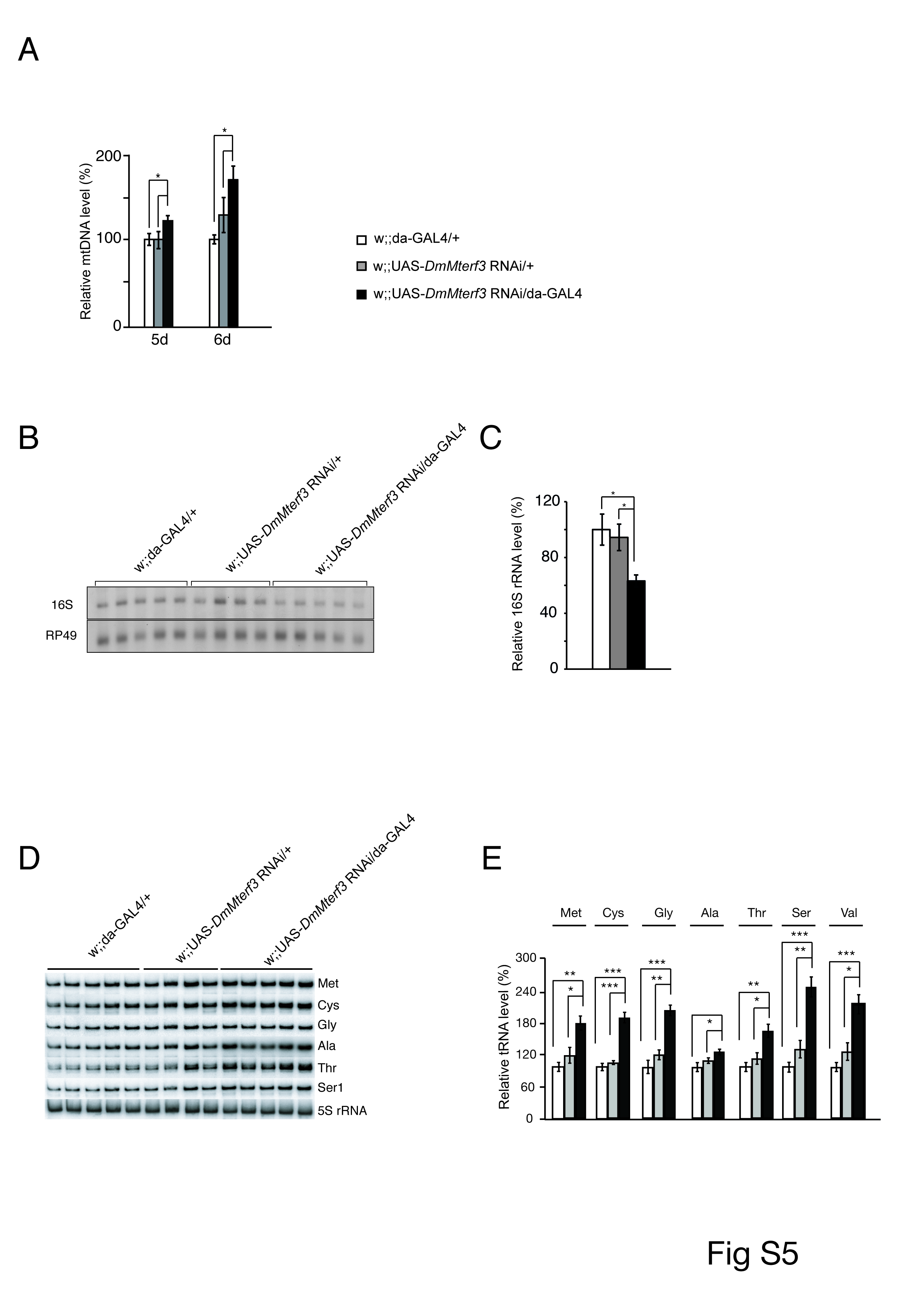

Supplement: Figure S5 — MtDNA and tRNA levels in DmMterf3 knockdown larvae. (A) Q-PCR analysis of mtDNA levels in control and DmMterf3 KD larvae 5 and 6 days ael. (B) Northern blot analysis of steady-state levels of mitochondrial large ribosomal RNA (16S rRNA) in control and DmMterf3 KD larvae at 3 days ael. Loading of gels was normalized to the transcript encoding the nuclear ribosomal protein 49. (C) Quantification of Northern blot analysis to assess steady-state levels of mitochondrial 16S rRNA in control and DmMterf3 KD larvae at 3 days ael. Loading of gels was normalized to the transcript encoding the nuclear ribosomal protein 49. (D) Northern blot analysis of steady-state levels of mitochondrial tRNAs in control and DmMterf3 KD larvae at 5 days ael. Loading of gels was normalized to nuclear 5S ribosomal RNA. (E) Quantification of Northern blot analysis to assess steady-state levels of mitochondrial tRNAs in control and DmMterf3 KD larvae at 5 days ael. Loading of gels was normalized to nuclear 5S ribosomal RNA. (TIF) [file pgen.1003178.s005.tif]

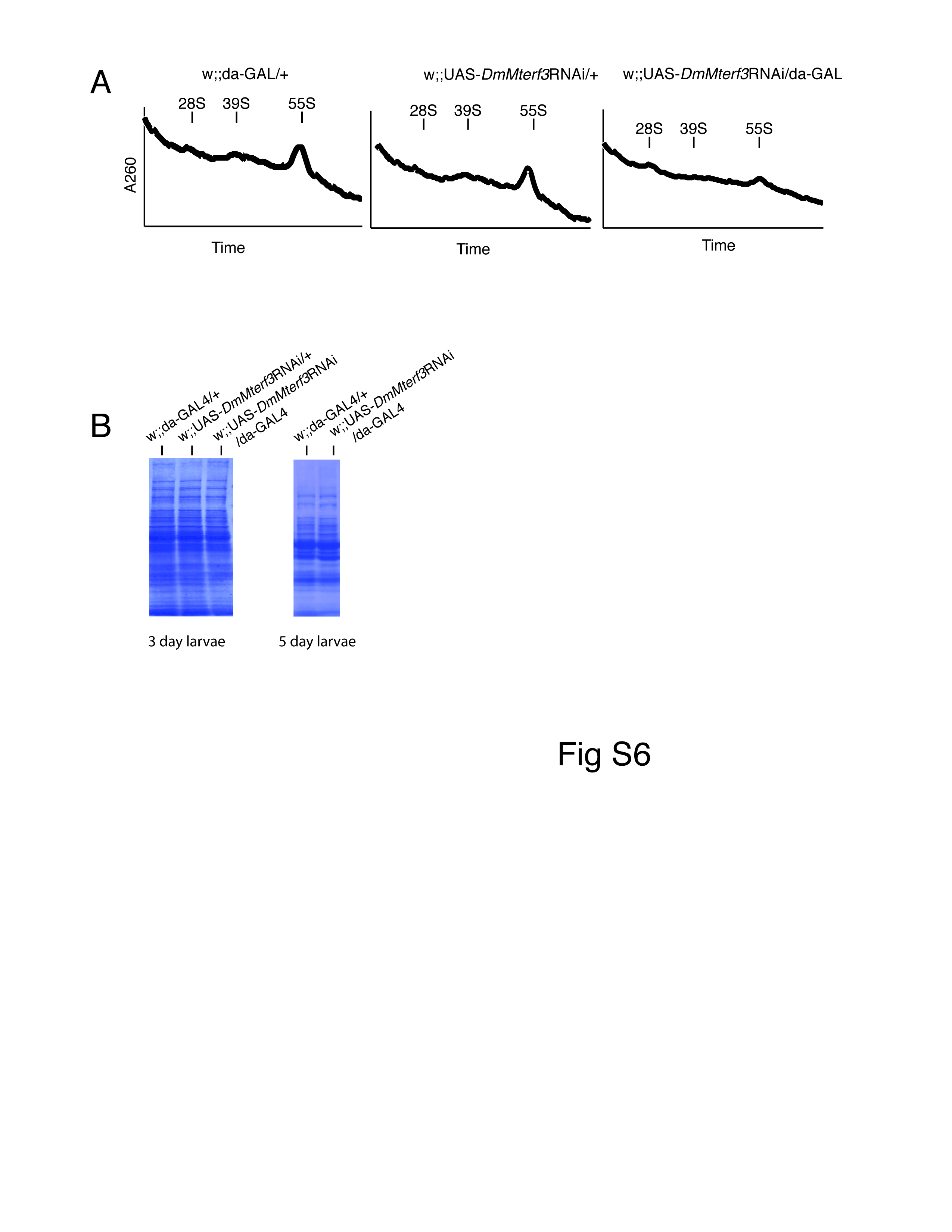

Supplement: Figure S6 — Sedimentation analysis of ribosomes in control and DmMterf3 knockdown flies. (A) Sedimentation analysis of ribosomal assembly in control and DmMterf3KD larvae at 3 days ael. The small (28S) ribosomal subunit, the large (39S) ribosomal subunit and the assembled (55S) ribosome were identified by the increased absorbance at 260 nm as indicated. (B) Aliquots of the mitochondrial protein extracts analyzed in (A) were separated by SDS-PAGE followed by Western blot analysis, using PVDF membranes, that were stained with Coomassie to assess loading. (TIF) [file pgen.1003178.s006.tif]

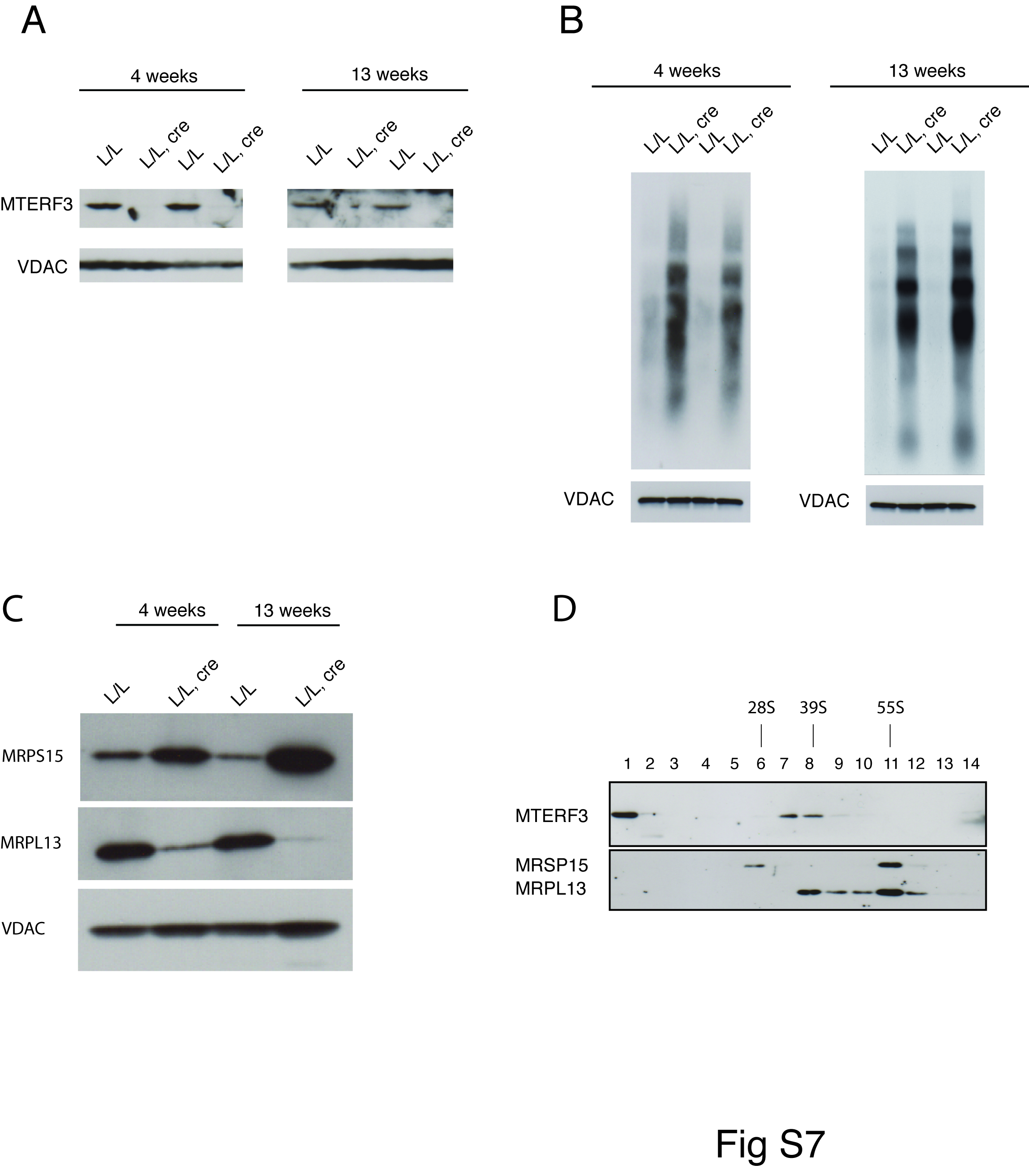

Supplement: Figure S7 — MTERF3 protein levels, de novo transcription and steady-state level of mitochondrial ribosomal proteins in control and Mterf3 knockout mouse hearts at 4 and 13 weeks of age. (A) Western blot analysis using an antibody against mouse MTERF3. VDAC is used as a loading control. (B) In organello transcription assays were performed in isolated crude mitochondrial preparations from control (L/L) and Mterf3 knockout (L/L, cre) mouse hearts at 4 and 13 weeks of age. VDAC is used as a loading control. (C) Western blot analysis of MRPL13 and MRPS15 steady-state protein levels in mitochondrial extracts from control (L/L) and Mterf3 knockout (L/L, cre) hearts at different ages. VDAC is used as a loading control. (D) Sedimentation analysis of the 28S, 39S, 55S and MTERF3 in wild-type heart mitochondria from 13-week-old mice, by centrifugation through a linear 10%–30% density sucrose gradient. The different fractions (numbered) were analyzed by SDS-PAGE and subsequent Western-blotting. (TIF) [file pgen.1003178.s007.tif]

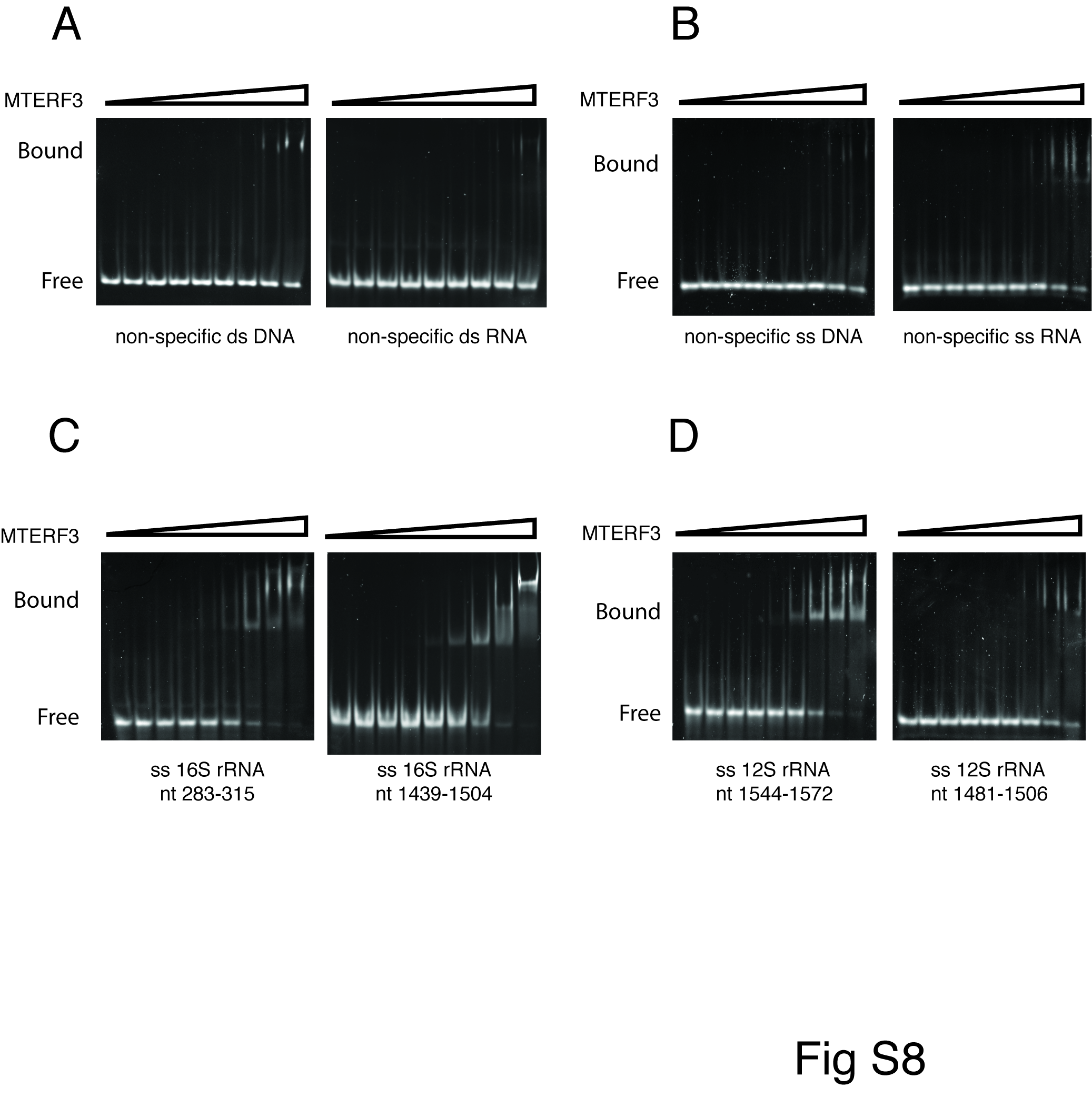

Supplement: Figure S8 — Binding of MTERF3 to DNA and RNA templates. (A) Binding of MTERF3 to double-stranded (ds) non-specific DNA or RNA. (B) Binding of MTERF3 to single-stranded (ss) non-specific DNA or RNA. (C) Binding of MTERF3 to mitochondrial 16S rRNA. (D) Binding of MTERF3 to mitochondrial 12S rRNA templates. The assays in (A–D) were performed with the following MTERF3 protein concentrations: 0, 0.02, 0.04, 0.08, 0.16, 0.36, 0.64, 1.28, 2.56 mM. The template amount was 40 ng for the experiments shown in (A–D). Free and bound templates are indicated. (TIF) [file pgen.1003178.s008.tif]

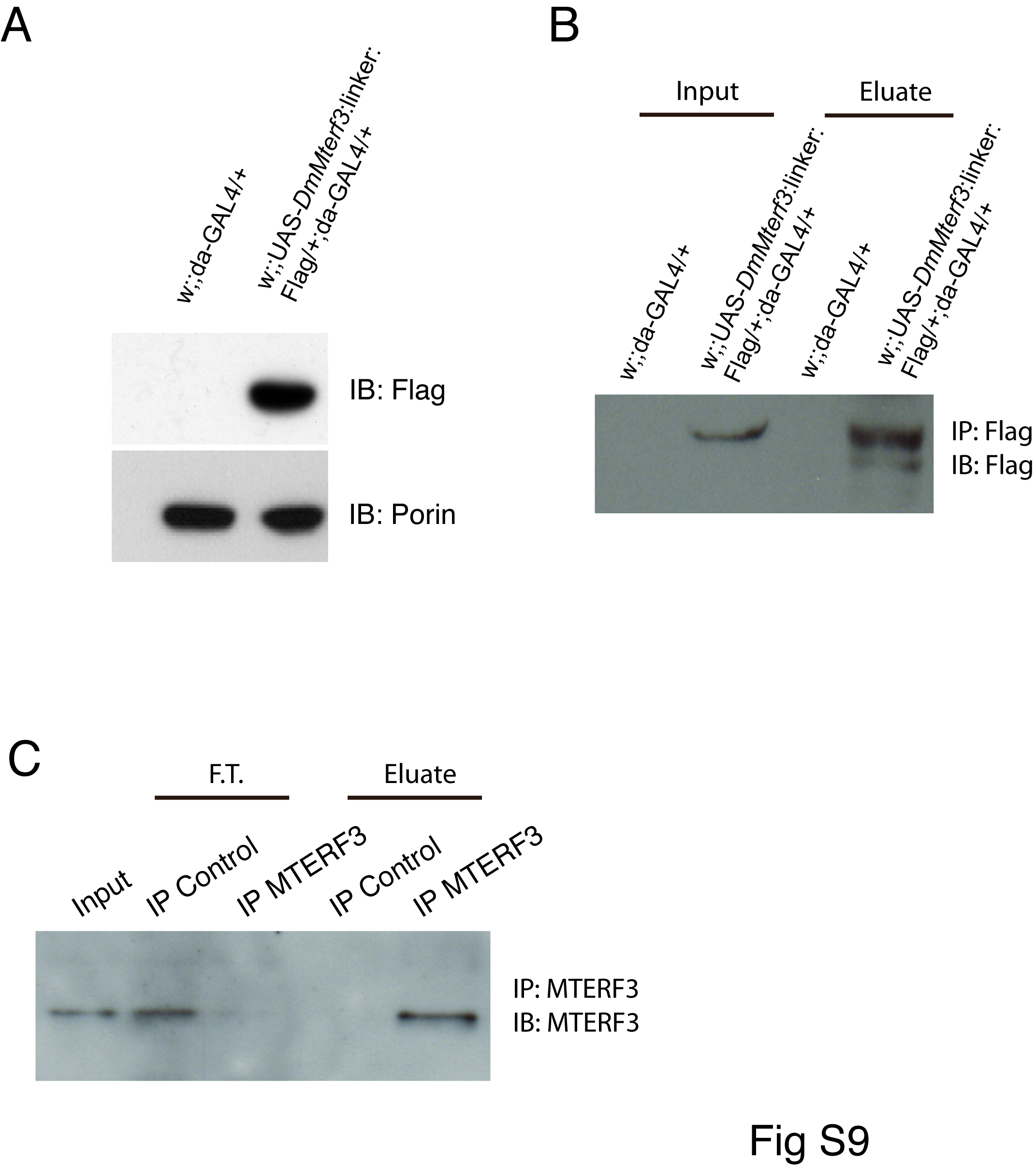

Supplement: Figure S9 — Stable expression of FLAG tagged DmMterf3 and MTERF3 immuno-precipitations. (A) Western blot analysis using an anti-Flag antibody (IB: Flag) on mitochondrial extracts from transgenic fly lines expressing DmMTERF3-linker-Flag. Porin is used as a loading control (IB: Porin). (B) DmMTERF3 was immuno-precipitated from Dm-MTERF3-linker-Flag-expressing flies using an anti-Flag resine (IP: Flag) and the same conditions as those used for RNA-IP. The different fractions were then blotted with an anti-Flag antibody (IB: Flag). (C) MTERF3 was immuno-precipitated (IP: MTERF3) from wild-type mouse heart mitochondria with the same conditions as those used for the RNA-IP. The different fractions were blotted with a monoclonal antibody directed against MTERF3 (IB:MTERF3). (TIF) [file pgen.1003178.s009.tif]
