# Supplementary material for: MTERF3 Regulates Mitochondrial Ribosome Biogenesis in Invertebrates and Mammals
Source: PLoS Genet. 2013 Jan 3;9(1):e1003178. doi: 10.1371/journal.pgen.1003178 (PMC3536695; doi:10.1371/journal.pgen.1003178)
Supplement: Table S1 — List of the primers used for cloning and qRT–PCR experiments. (DOC) [file pgen.1003178.s010.doc]

|  | **Rescue experiments** |  |
| --- | --- | --- |
| **ET_Dp_F** | ccctactatatgcttttctgtagtttcttcgctgttagcattcacttcgcagatctcacaacatacgagccggaagcata | Bgl II |
| **ET_Dp_R** | aggcggactgattgctgctgatgggtggtgtggtggtggctcaaatacatgcggccgcatgtgcgcggaacccctatttg | Not I |
|  | **Generation of MTERF3 mutant** |  |
| **ET_5’arm_F** | tttgcttttgccagggaggagacaaacagaatgcaggcaatgaagattgggcatgccacaacatacgagccggaagcata | SphI |
| **ET_5’arm_R** | ttctgttgacttttgaaaatatatgcatatgcatatatgaatatgaaaagccgcggatgtgcgcggaacccctatttg | SacII |
| **ET_3’arm_F** | aactttggcagccaactgctggtcacattcgcgacatctggcaacgcctcggcgcgcccacaacatacgagccggaagcata | AscI |
| **ET_3’arm_R** | atgattaaaaagtatgatttcatcgaaagttttttggcgataagggacttaggcct atgtgcgcggaacccctatttg | StuI |
| **PCR_primer1** | tttggatccttacttatcatcatcatccttataatctctcgttttcaaaaacaaatc |  |
| **PCR_primer2** | ccttccgtatgtctgcctgt |  |
| **PCR_primer3** | cttgaagggacccatgagaa |  |
|  |  |  |
|  | **Quantitative RT-PCR (SYBR)** |  |
| **16S_F** | acctggcttacaccggttt |  |
| **16S_R** | gggtgtagccgttcaaattt |  |
| **12S_F** | gataacgacggtatataaactgattaca |  |
| **12S_R** | gaggaacctgttttttaatcga |  |
|  |  |  |
|  | **Northern blot analyses** |  |
| **ND2_F** | cttggttaggagcttgaataggt |  |
| **ND2_R** | aaatggaggtaatcctcctaatga |  |
| **ND5_F** | atccaaataaaccccctacaa |  |
| **ND5_R** | tcgaattggggatgtagctt |  |
| **ND4_F** | tggagcttcaacatgagcttt |  |
| **ND4_R** | tgaggttatcagccagaacg |  |
|  | **DmMterf3-GFP** **construct** |  |
| **F** | tgtaaaacggtcgacatgttttgttcagctcacg | SalI |
| **R** | ctatgacccgggtctcgttttcaaaaacaaatc | SmaI |
|  | **DmMterf3-linker-FLAG** |  |
| **F** | agcagcagcagctcctctcgttttcaaaaacaaatc |  |
| **R** | ggagctgctgctgctggagactacaaggacgatgacgac |  |
