# Supplementary material for: MTERF3 Regulates Mitochondrial Ribosome Biogenesis in Invertebrates and Mammals
Source: PLoS Genet. 2013 Jan 3;9(1):e1003178. doi: 10.1371/journal.pgen.1003178 (PMC3536695; doi:10.1371/journal.pgen.1003178)
Supplement: Text S1 — Supporting materials and methods including supporting references. (DOC) [file pgen.1003178.s011.doc]

**Text S1: Supplemental materials and methods**

Drosophila stocks and maintenance

Knockdown (KD) studies were performed using the UAS-*DmMterf3* RNAi line denoted w;;UAS-*DmMterf3*RNAi (#26692) from the Vienna Drosophila RNAi Center. Ubiquitous *DmMterf3* KD was achieved by crossing the UAS-*DmMterf3* RNAi line to the daughterless-GAL4 (da-GAL4) driver line. The UAS-RNAi line and the da-GAL4 driver lines were backcrossed for at least 6 generations to the white Dahomey background (wDahT). All fly stocks were free from the endosymbiotic bacterium *Wolbachia*. Flies were propagated and experiments were conducted at 25°C on a 12h:12h light:dark cycle at constant humidity on a standard sugar-agar-yeast medium.

Bioinformatics

Sequences were searched using PSI-BLAST [1] against the NCBI RefSeq protein database. Subfamilies were aligned individually using ClustalX [2] and the alignment of all four subfamilies was done manually using structural information from MTERF1 [3] and MTERF3 [4]. Phylogenetic analysis was done on the full-length sequences using PhyML [5] with default parameters and 500 iterations. Display of the tree was done in Illustrator (Adobe). Nucleotide and polypeptide sequences were aligned using Geneious v5.5 software (Biomatters Ltd) with the software’s default settings.

Co-localisation studies

Full-length *DmMterf3* cDNA was obtained from the Drosophila Genomics Resource Center (LD27042). Two amino acid changing substitutions were identified in the *DmMterf3* cDNA in comparison with the reference sequence (FBtr0081087). The corresponding mutations at nucleotide positions 415 and 710 were changed by site-directed mutagenesis of the cDNA, using the QuickChange II XL Site-Directed Mutagenesis Kit (Agilent Technologies). *DmMterf3* cDNA was cloned into the plasmid pAcGFP1-N2 (Clontech) to generate the vector p*DmMterf3*-FLAG-AcGFP1, which encodes a fusion protein consisting of *DmMterf3* with an in-frame addition of green fluorescent protein (GFP) at its carboxy-terminus (DmMTERF3-FLAG-GFP). Schneider 2R+ and HeLa cells were transfected as previously described [6]. Mitochondria were counter-stained with 100nM MitoTracker Deep Red FM (Invitrogen). Live cell image acquisition was performed with a Leica TCS SP5-X confocal microscope (Leica Microsystems) in sequential scanning mode. The co-localisation rate was determined by the software LAS AF (Leica Microsystems) under the following conditions and using the following calculations: threshold 30%, background 20%, co-localisation rate [%] = co-localisation area/area foreground, and area foreground = area image − area background.

Western blot analysis

In flies, Western blot analyses were performed using whole larvae or mitochondrial protein extracts according to the Cell Signalling Technology protocol. Primary antibodies were: complex V (Mitoscience MS504, dilution 1:5000), Porin (Mitoscience MSAO3, dilution 1:2000), actin (Sigma, dilution 1:1000) and Flag M2 (Sigma, dilution 1:500). Visualization was performed with ECL Western blotting reagents (Bio-Rad). In mice, Western blot analyses were conducted on heart mitochondrial extracts. VDAC antibody was purchased from Calbiochem. Polyclonal antiserum against mouse MRPS15 was generated in rabbits by using a mixture of two peptides (N-KILRQTNYDVFEKTC-C and N-CPENPSNAVPEKTQVN-C). MRPL13 antiserum was provided by Dr Linda Spremulli. The monoclonal antibody directed against mouse MTERF3 was generated by Dr Elisabeth Kremmer using the purified N-his-MTERF3 recombinant protein.

DNA isolation and Q–PCR

DNA from fly larvae was extracted using the DNAeasy Kit (Qiagen). Mitochondrial DNA levels were determined by quantitative real-time PCR (Q-PCR) on a 7900HT Real Time PCR system (Applied Biosystems), using SYBR green master mix (Invitrogen). Reactions were carried out in triplicates per sample in a final volume of 12µl with 5ng of DNA and 10pmol of specific primers.

RNA isolation, QRT-PCR, and Northern blot analysis

Total RNA from Drosophila larvae was extracted using the Totally RNA KIT (Ambion). Reverse transcription and subsequent q-PCR was performed using the High capacity RNA-to-cDNA kit (Applied Biosystems) and the Taqman 2x Universal PCR mastermix, No Amperase UNG (Applied Biosystems), respectively. Custom-made TaqMan probes against Drosophila mitochondrial transcripts were obtained from Applied Biosystems and are listed in Table S1. For Northern blot analysis, RNA was fractionated in 1.2% agarose gels and blotted to Hybond-N+ membranes (GE Healthcare). Membranes were hybridized with ^32^P-labeled probes in RapidHyb (GE Heathcare), washed and exposed to PhosphoImager Screens and/or X-ray films. Labelling of mitochondrial double-stranded DNA probes and oligonucleotides was performed as described in [6]. Primers and oligonucleotides used for Northern blot are listed in Table S1.

Biochemical evaluation of respiratory chain function

Isolation of mitochondria from third-instar larvae was performed as described [6] but with modifications in buffer composition. Briefly, fly larvae were washed and gently homogenized in ice-cold MSB buffer (210mM mannitol, 70mM sucrose, 10mM EDTA, 50mM Tris, pH 7.5) using 15ml Dounce homogenizers. Protein concentration was determined using Bradford assay (BioRad) and aliquots corresponding to 10µg mitochondrial proteins were pelleted and resuspended in resuspension buffer (250mM sucrose, 15mM K2HPO4, 2mM MgAc2, 0.5mM EDTA and 0.5g/L HSA, pH 7.2). Biochemical activities of respiratory chain complexes were determined as described in [7].

Blue native-polyacrylamide gel electrophoresis (BN-PAGE) and in-gel enzyme activity

For BN-PAGE, 75µg of mitochondria were pelleted and disrupted in 50µl ice-cold digitonin buffer (1% digitonin, 20mM Tris pH 7.4, 0.1mM EDTA, 50mM NaCl, 10% glycerol, 1mM PMSF). After 15 min incubation on ice, non-solubilized material was removed by centrifugation at 4°C and the supernatant mixed with 5µl of 10x loading dye (5% (w/v) Coomassie Brilliant Blue G-250, 100mM Tris pH 7.0, 500mM 6-aminocaproic acid) and loaded on 4–10% gradient BN-PAGE gels [8]. In-gel complex I activity was determined by incubating the BN-PAGE gels in 2mM Tris-HCl pH 7.4, 0.1mg/ml NADH (Roche) and 2.5mg/ml iodonitrozolium (Sigma). In-gel complex IV activity was determined by incubating the BN-PAGE gels in 0.05M phosphate buffer pH 7.4, 25mg 3,3-diaminobenzidine tetrahydrochloride (Sigma), 50mg cytochrome c (Sigma), 3.75g sucrose and 1mg catalase (Sigma).

**Gel mobility shift assay (EMSA)**

Templates were either double- or single-stranded DNA or RNA oligonucleotides. The reactions were performed in a 20μl volume containing 20mM Tris–HCl, pH 8.0, 10mM MgCl_2_, 60mM NaCl, 15% glycerol, 1mM EDTA, 0.1 mg/ml BSA, 1.0mM DTT, 40ng of template and varying amounts of purified human recombinant MTERF3, as specified in the figure legend. MTERF3 was purified as previously described [4]. The samples were incubated at room temperature for 15 min and separated by electrophoresis in a 6% DNA retardation gel (Invitrogen) in 0.5x TBE. The gel was stained with SYBR Gold (Invitrogen) according to the manufacturer’s instructions. Bands were visualized using the Gel Doc EZ system (Bio-Rad).

Statistical analysis

Data were presented as mean ± SEM. Unpaired t-test was used to analyze all data statistically.

**References**

1. Altschul SF, Madden TL, Schaffer AA, Zhang J, Zhang Z, et al. (1997) Gapped BLAST and PSI-BLAST: a new generation of protein database search programs. Nucleic Acids Res 25: 3389-3402.

2. Higgins DG, Thompson JD, Gibson TJ (1996) Using CLUSTAL for multiple sequence alignments. Methods Enzymol 266: 383-402.

3. Yakubovskaya E, Mejia E, Byrnes J, Hambardjieva E, Garcia-Diaz M (2010) Helix unwinding and base flipping enable human MTERF1 to terminate mitochondrial transcription. Cell 141: 982-993.

4. Spahr H, Samuelsson T, Hallberg BM, Gustafsson CM (2010) Structure of mitochondrial transcription termination factor 3 reveals a novel nucleic acid-binding domain. Biochem Biophys Res Commun 397: 386-390.

5. Guindon S, Delsuc F, Dufayard JF, Gascuel O (2009) Estimating maximum likelihood phylogenies with PhyML. Methods Mol Biol 537: 113-137.

6. Bratic A, Wredenberg A, Gronke S, Stewart JB, Mourier A, et al. (2011) The bicoid stability factor controls polyadenylation and expression of specific mitochondrial mRNAs in Drosophila melanogaster. PLoS Genet 7: e1002324.

7. Wibom R, Hagenfeldt L, von Dobeln U (2002) Measurement of ATP production and respiratory chain enzyme activities in mitochondria isolated from small muscle biopsy samples. Anal Biochem 311: 139-151.

8. Schagger H, von Jagow G (1991) Blue native electrophoresis for isolation of membrane protein complexes in enzymatically active form. Anal Biochem 199: 223-231.
